# Forma mentis networks map how nursing and engineering students enhance their mindsets about innovation and health during professional growth

**DOI:** 10.7717/peerj-cs.255

**Published:** 2020-03-02

**Authors:** Massimo Stella, Anna Zaytseva

**Affiliations:** 1 Complex Science Consulting, Lecce, Italy; 2 i4Helse, University of Agder, Grimstad, Norway

**Keywords:** Complex networks, Stance detection, Psycholinguistics, Education, Learning outcomes, Healthcare, Mindset modelling, STEM, Attitude, Entropy

## Abstract

Reconstructing a “forma mentis”, a mindset, and its changes, means capturing how individuals perceive topics, trends and experiences over time. To this aim we use forma mentis networks (FMNs), which enable direct, microscopic access to how individuals conceptually perceive knowledge and sentiment around a topic, providing richer contextual information than machine learning. FMNs build cognitive representations of stances through psycholinguistic tools like conceptual associations from semantic memory (free associations, i.e., one concept eliciting another) and affect norms (valence, i.e., how attractive a concept is). We test FMNs by investigating how Norwegian nursing and engineering students perceived innovation and health before and after a 2-month research project in e-health. We built and analysed FMNs by six individuals, based on 75 cues about innovation and health, and leading to 1,000 associations between 730 concepts. We repeated this procedure before and after the project. When investigating changes over time, individual FMNs highlighted drastic improvements in all students’ stances towards “teamwork”, “collaboration”, “engineering” and “future”, indicating the acquisition and strengthening of a positive belief about innovation. Nursing students improved their perception of ‘robots” and “technology” and related them to the future of nursing. A group-level analysis related these changes to the emergence, during the project, of conceptual associations about openness towards multidisciplinary collaboration, and a positive, leadership-oriented group dynamics. The whole group identified “mathematics” and “coding” as highly relevant concepts after the project. When investigating persistent associations, characterising the core of students’ mindsets, network distance entropy and closeness identified as pivotal in the students’ mindsets concepts related to “personal well-being”, “professional growth” and “teamwork”. This result aligns with and extends previous studies reporting the relevance of teamwork and personal well-being for Norwegian healthcare professionals, also within the novel e-health sector. Our analysis indicates that forma mentis networks are powerful proxies for detecting individual- and group-level mindset changes due to professional growth. FMNs open new scenarios for data-informed, multidisciplinary interventions aimed at professional training in innovation.

## Introduction

“Forma mentis is a Latin expression indicating the mindset of an individual or a population towards a given entity. For instance, how do people structure their mindsets around the concept of “climate change”? Quantifying people’s forma mentis or perception of a specific topic represents an open challenge, especially in absence of human intervention (Jaffe, 2009). In computer science and psycholinguistics this problem of capturing people’s attitudes and mindsets is known as *stance detection* and it is commonly based on linguistic data (Jaffe, 2009; Mohammad et al., 2016). Several independent studies have confirmed that word features and associations are, in fact, highly predictive of a given positive, neutral or negative stance towards a topic (Mohammad et al., 2016; Balahur et al., 2018; Sun et al., 2018). This is not surprising. Language use, with its meanings as encapsulated within and between concepts and their associations, can drastically change across individuals and thus enable the recognition of different stances. Aitchison condensates this variability of conceptual associations and usage by saying that: “Word meanings are like stretchy pullovers, whose outline contour is visible, but whose detailed shape varies with use” (Aitchison, 2012).

With the advent of Big Data, the availability of large-scale datasets and the rise of new tools for language modelling and analysis like cognitive network science (Siew et al., 2019), stance detection has been successfully achieved also in absence of human coding (Balahur et al., 2018; Mohammad, Sobhani & Kiritchenko, 2017). The automatic manipulation of linguistic data is usually tackled from various directions. Machine learning, among other techniques, has proven quite powerful in classifying the stance of short texts from social media (Mohammad et al., 2016; Mohammad, Sobhani & Kiritchenko, 2017; Balahur et al., 2018; Sun et al., 2018), although often lacking any insight about the complex reasons and circumstantial conditions behind the nuances of a given attitude or stance.

This “black-box nature of machine learning approaches to stance detection can be particularly problematic when one is not interested only in a single stance classification (e.g., a cop perceives violence as negative) but rather in the analysis of how a set of correlated stances can change over time (e.g., a group of cops changing their perceptions of “violence, “guns” and “law” over time). Changes in a given mindset need to be analysed together with as much contextual information as possible in order to achieve meaningful interpretations (Jaffe, 2009). Modifications of a given stance might be possible due to a variety of reasons (Eagly & Chaiken, 1995), which could be classified mainly in internal causes (e.g., personal growth leading naturally to a different perception of violence over time) and external causes (e.g., learning new elements, understanding or experiencing something can change the way one perceives violence). Furthermore, mindsets, perceptions and attitudes are functions of both knowledge and affect components (Edwards, 1990; Jaffe, 2009; Boiger, De Deyne & Mesquita, 2013), so that also their changes have to be described in terms of modifications of semantic knowledge and affect.

In this manuscript we focus on changes in the mindset of a small population of individuals that are mainly due to experiential learning through an intensive professional training offered during a short span of time. In order to detect changes in the mindsets of individuals we use the recent approach by Stella et al. (2019), who tackled stance detection by introducing the framework of *forma mentis networks*. These quantitative tools are based on the well-documented finding that the way individuals associate and perceive distinct concepts in a cognitive system, usually called *mental lexicon* (Aitchison, 2012), can be highly informative of a wide variety of cognitive processes like language processing (cfr. also Siew et al., 2019), spoken word recognition (Vitevitch, Siew & Castro, 2018), early word learning (Stella, Beckage & Brede, 2017; Sizemore et al., 2018) and, more importantly, of writing styles (Akimushkin, Amancio & Oliveira Jr, 2018). The mental lexicon can also provide information about creativity (Kenett, 2019; Stella & Kenett, 2019), personality traits such as openness to experience (Christensen et al., 2018) and curiosity (Lydon-Staley et al., 2019), and knowledge structuring (Thomas & Zaytseva, 2016; Stella, Ferrara & De Domenico, 2018). Forma mentis networks build on the above results by capturing how individuals structure their knowledge and perceive its affective components (Stella et al., 2019). The combination of these two elements makes forma mentis networks informative not only about the global stance of individuals towards a given topic but also about the microscopic associations connecting positive/negative/neutral concepts. The simultaneous presence of macroscopic assessments and microscopic information, absent in other quantitative approaches powered by machine learning Mohammad, Sobhani & Kiritchenko (2017) or by semantic network models for discourse analysis Leifeld (0000); Stella, Ferrara & De Domenico (2018), can be informative on the circumstances driving potential attitude changes, and it represents the main motivation for the adoption of forma mentis networks in this study.

In order to assess the power of forma mentis networks in highlighting attitude changes pre- and post intervention, we here focus on a specific case study. We focus on how medical and engineering students perceived innovation within the Norwegian e-health sector before and after a summer job experience for i4Helse and the Young Industrial Innovators at Universitetet i Agder, Norway.

The choice of this case study is highly advantageous. On the one hand, this event represented an intensive training happening on a short span of time, and it thus reduced the influence of personal growth over attitude change, with the benefit of making it easier to relate key changes in the individual mindsets mainly to experiential learning (Edwards, 1990; Eagly & Chaiken, 1995). On the other hand, the detection of potential “improvements” in the perception of innovation within the mindset of healthcare (nursing, to be specific) and engineering students can offer useful knowledge for facilitating the development of entrepreneurial attitudes of relevance for the innovation sector. Notice that by “improvement” we here mean the strengthening of a positive emotional stance towards a given topic, featuring also more specific, concrete and skill-focused concepts rather than generic ideas.

Health and welfare belong to highly complex domains, where expertise implies in-depth professional knowledge in medicine, biology of human body and lifestyle patterns, as well as knowledge in health policy, law, budget and labour management (Zaytseva, 2016). Moreover, the nature of health- and homecare domains sets high requirements for health- and homecare professionals, like being exceptionally empathetic and sensitive to human needs, rather than “aggressive in gaining new knowledge, learning and competing (which is the case for high-tech professionals). The labour market of Norway is in increasing demand for more professionals in nursing, with skills and knowledge in technology (Zaytseva, 2016). Hence, the investigation of the evolution of the mindsets of interacting nursing and engineering students represents a challenge to the silo-driven educational system persisting in Norway  Thistlethwaite (2012) and Zaytseva (2016), which educates future professionals to work within only one domain of practice, thus making it hard for these trainees to adapt to the introduction of new methods, especially in the cross-disciplinary Norwegian expanding field of e-health. In this way, the exploration of changes in the cognitive perception of health- and homecare students (as future professionals) represents an innovative experimental *testing environment* for assessing the overcoming of a silo-driven education Thistlethwaite (2012) and the development of inter-professional and communicative skills of critical relevance for the job market Begat, Ellefsen & Severinsson (2005), Thistlethwaite (2012), Zaytseva (2016) Thomas & Zaytseva (2016) of emerging technologies, health and welfare.

Within this challenging scenario, accessing the students’ mental perception of innovation before and after the summer job experience provides a data-informed model about specific learning aspects that can be improved, built, restructured or already positively perceived and linked to enhanced professional growth. In this way, the manuscript adopts mainly two tightly interconnected research questions. The first one is relative to the capability for forma mentis networks to detect and highlight changes in the students’ mindsets before and after their summer job experience. The subsequent research question is characterising the nature of such potential changes in mindsets, identifying which modifications were related to the considered intervention in nursing and engineering students.

This manuscript has the following structure. The ‘Methods’ section outlines how forma mentis networks combine semantic knowledge (e.g., the presence/absence of specific conceptual associations) and affect patterns (e.g., how individual concepts are positively/negatively/neutrally perceived and also associated). That section reports also on the students who participated in the experiment. The ‘Results’ section is divided in three different subsections: the first two outline changes in the forma mentis networks at the individual and group levels, respectively, while the last subsection focuses on identifying the key common, stable aspects of the global mindset of students. The manuscript ends with a discussion of the main strengths and limitations of our approach and the resulting conclusions.

## Methodology

### Participants

This study targeted college students hired for the Young Industrial Innovators, partnered with the i4Helse centre, located at the campus Grimstad of the University of Agder, Norway. Eight students from healthcare science, engineering and mechatronics were hired in order to build a small robot serving as a personal assistant for tracking patients’ vitals in hospitals (see also Text S1 for more details). A small group of *N* = 6 students voluntarily enrolled in the current cognitive experiment. Participants had a median age of 24 years, three of them were male students studying engineering and mechatronics while three of them were female healthcare students practising as nurses. All the interviewed students were native Norwegian speakers.

The research protocol was designed in accordance to the ethics indications provided in the Declaration of Helsinki. Written informed consent was obtained from all subjects after a clear explanation of the research methodology. Individual privacy and anonymity were protected, the data was gathered in a fully anonymous way. No sensitive data, allowing for a profiling of the original identity of each individual participant, was either gathered or stored. This study received IRB approval by the University of Agder with reference number RITM0054729.

### Forma mentis networks

Forma mentis networks (FMNs) were recently introduced by Stella and colleagues in Stella et al. (2019). These complex networks are representations of conceptual knowledge containing two types of psycholinguistic data: (i) free associations between concepts (i.e., associations indicating which concepts come to mind to an individual when reading a given cue words), and (ii) conceptual valence of individual words (i.e., labels assessing how positive/negative/neutral a given concept is perceived by an individual). Free associations constitute a network of links between concepts, representative of the semantic knowledge of individuals (De Deyne, Navarro & Storms, 2013; Kenett et al., 2017; Kenett, 2019), while valence labels reconstruct affect patterns, representative of the sentiment towards individual concepts (Kuperman et al., 2014). The combination of these two elements in a forma mentis network constitutes a networked, quantitative representation of an individual’s stance towards the selected cue words (Stella et al., 2019).

In here, we selected Norwegian cues related to professional growth and development in relation to the healthcare sector. In order to obtain large, densely connected networks, we recurred to a continuous free association game (De Deyne, Navarro & Storms, 2013) based on 75 cues, with participants attributing up to three associates to each cue according. Participants were instructed to leave blank spaces in case nothing came up to their minds. Valence labels were encoded as “pos”/“neg”/“neu” default values for positive, negative and neutral concepts, respectively. The experiment was performed in Norwegian. Cue words are listed in the Supplementary Information.

Every student was interviewed at the beginning and at the end of their summer job experience, thus providing two snapshots of their perception of the healthcare sector. Students were also invited to provide feedback on their experience at the end of the summer job but without having access to the quantitative results reported here in this manuscript (see also Text S2 for more details).

Individual and global FMNs were analysed. Individual networks were built by considering the students’ replies in Norwegian. Self-evident typos or different spellings of the same lexical item were manually corrected (e.g., “fremtid” was changed to “framtid”). Responses with ambiguous typos were discarded. No additional lemmatisation or stemming was applied to the data. Global forma mentis networks were built by aggregating together all students’ associations. In these global networks, emotional labels were reconstructed by means of a consensus rule. The label of a concept evaluated by *x* students was determined as the valence attributed by at least “half of x + 1” students. For instance, “internett” was labelled by a total of *x* = 4 students, three of them labelled internet as “positive” and one of them as “neutral”. In the global network, “internett” was labelled as “positive” because at least *x*∕2 + 1 = 3 students perceived it as positive. In case no clear consensus among perceptions is achieved, the concept is labelled as neutral. For instance, “forvente” (to expect) was labelled by *x* = 6 students, with two of them perceiving it as “negative”, three as “neutral” and one as “positive”. No label appeared more than *x*∕2 + 1 = 4 times in these labelling. Without such consensus, “forvente” was labelled as neutral in the global forma mentis network. This consensus rule makes the global forma mentis network selective in determining which concepts are positively or negatively perceived by most individuals in the considered group.

### Graph distance entropy and cartography

Network cartography is the representation of quantitative information describing nodes and their relevance in a given network structure. For examples and applications see for reference (Guimera & Amaral, 2005; Lommi & Koponen, 2019; Stella et al., 2018; Stella & De Domenico, 2018). With the aim of identifying words of relevance in a cognitive network of conceptual associations, we adopt the cartography based on graph distance entropy and closeness from Stella & De Domenico (2018), which was found to highlight relevant concepts from learning environments.

Distance entropy cartography is based on closeness centrality and on graph distance entropy. Closeness centrality represents the inverse of the mean value of the distribution of path lengths from a given node to the rest of the connected network (see Newman, 2018). In formulas, if *d*_*ij*_ is the network distance between nodes *i* and *j*, i.e., the smallest number of links connecting *i* and *j*, then closeness centrality for node *i* is defined as:

(1) \begin{eqnarray*}c(i)= \frac{N}{\sum _{j=1}^{N}{d}_{ij}} ,\end{eqnarray*}

where *N* is the number of nodes connected to *i*. Closeness centrality is not a good estimator of relevance in disconnected networks (Newman, 2018). In our case closeness was applied to the largest connected component of the core forma mentis networks since the remaining lexical islands/disconnected components included 4 or less concepts. From a cognitive perspective, closeness captures how information and spreading activation signals can uniformly spread across a given network topology. Several studies have shown the predictive power of closeness centrality for modelling a variety of cognitive phenomena related to early word acquisition (Stella, Beckage & Brede, 2017; Siew et al., 2019; Stella, 2019) and language processing in semantic memory (Kenett et al., 2017; Goldstein & Vitevitch, 2017; Vitevitch, Siew & Castro, 2018; Castro & Stella, 2019).

Closeness itself is an average value, equivalent to the inverse of the average distance between one node and all others connected to it. Hence, closeness does not provide info about the variance or spread of the distribution of distances between nodes. This sort of error bound is captured by *distance entropy h*(*i*), defined by  Stella & De Domenico (2018) as the Shannon entropy of the set **d**^(*i*)^ ≡ (*d*_*i*1_, …, *d*_*ij*_, …, *d*_*iN*_) of distances between *i* and any other node *j* connected to it (1 ≤ *j* ≤ *N*). If *M*_*i*_ and *m*_*i*_ are the maximum and minimum values, respectively, that network distance can assume, then graph distance entropy is defined as:

(2) \begin{eqnarray*}h(i)=- \frac{1}{\text{log}({M}_{i}-{m}_{i})} \sum _{k=1}^{{M}_{i}-{m}_{i}}{p}_{k}^{(i)}\text{log}{p}_{k}^{(i)},\end{eqnarray*}

where *p*_*k*_ is the probability of finding a distance equal to the integer value *k*. By definition *h*(*i*) ranges between 0 and 1. The minimum value 0 is relative to a node having the same distance to all other nodes connected to it. This can happen only for the centres of a star graph, which are at distance *k* = 1 from all other nodes. Hence, for star centres the distribution of distances has no spread or variance and *h*(*i*) = 0. For nodes in infinite regular lattices, all network distances are equally possible and such case of largest variance corresponds to the highest entropy possible, *h*(*i*) = 1. Hence, graph distance entropy can be seen as a metric determining how close to a star graph or to a regular lattice the connections of a given node are. In general, the lower the entropy, the closer the node is to a configuration of centrality equivalent to a star centre (Stella & De Domenico, 2018). From a cognitive perspective, lower values of graph distance entropy were found to efficiently identify words of relevance in learning environments represented as cognitive networks of conceptual associations (Stella & De Domenico, 2018).

In this work, we aim at using combinations of closeness centrality (values closer to 1 identify relevant nodes) and distance entropy (values closer to 0 identify relevant nodes) for identifying central nodes in the *core forma mentis network* of students. We define such network as the resulting FMN obtained with conceptual associations present both at the start and at the end of the intervention, i.e., conceptual associations that did not change over time. The core networks included a largest connected component with 196 nodes and 244 edges and an ensemble of 8 smaller connected components/lexical islands constituted by 5 nodes on average. We focused our attention on the largest connected component and investigated its distance cartography: We considered a scatter-plot where each point represents a node and its coordinates are, respectively, closeness centrality and graph distance entropy. We then considered quartiles for the distributions of closeness and distance entropy and classified nodes accordingly. By definition, relevant nodes had to be in the upper quartile of the closeness distribution and in the lower quartile of the distribution of graph entropy. This approach allowed to combine multiple properties of conceptual associations and conceptual distances (for a definition of semantic distance cfr. Kenett et al., 2017), leading to a more robust identification of relevant nodes even in a relatively small network of a couple of hundred of nodes, building upon the previous testings made in Stella & De Domenico (2018).

## Results

This section reports on the quantitative results observed in the forma mentis networks that were built upon either individual students or groups of individuals (e.g., all the actual participants). The outline of our quantitative results follows three steps. We start with the microscopic identification of changes in the individual forma mentis of students pre- and post-intervention and identify which concepts related to engineering, soft skills and healthcare underwent the most dramatic changes in terms of conceptual associations and emotional perception. We then aggregate individual networks into a global forma mentis network arising from a consensus on how individual students perceive concepts. We detect the most drastic changes in the global structure of these networks and highlight how collectively the whole group of students changed stances towards engineering, soft skills and healthcare. While the first two steps focus on perceptual changes, the third step characterises those conceptual associations that remained stable pre- and post-intervention. We describe these persistent links in terms of a *core* forma mentis network, characterising the global, collective mindset of students during the whole event. We use this resilient structure in order to characterise and profile the key features of the students’ mindset through network and entropy-based measures of cognitive relevance.

## Longitudinal Comparisons Over Time Highlight Knowledge and Stance Restructuring

A longitudinal comparison allows to highlight potential changes in the mindsets of individual students before and after their personal experience with the intervention, i.e., the summer job event. At the individual level, minor fluctuations in the layout of associations are expected (De Deyne, Navarro & Storms, 2013; Kenett et al., 2017), since the free association game is not constrained by specific rules in the recollection of words (i.e., there is no unique or wrong or correct answer to each cue). However, many independent studies agreed on defining as “strong” those free associations made by at least two different individuals (De Deyne, Navarro & Storms, 2013; Kenett et al., 2017). Building upon this, we focus here on analogous structural changes in the forma mentis networks repeated at least once either by the same individual over time or by at least two different individuals.

**Figure 1:**
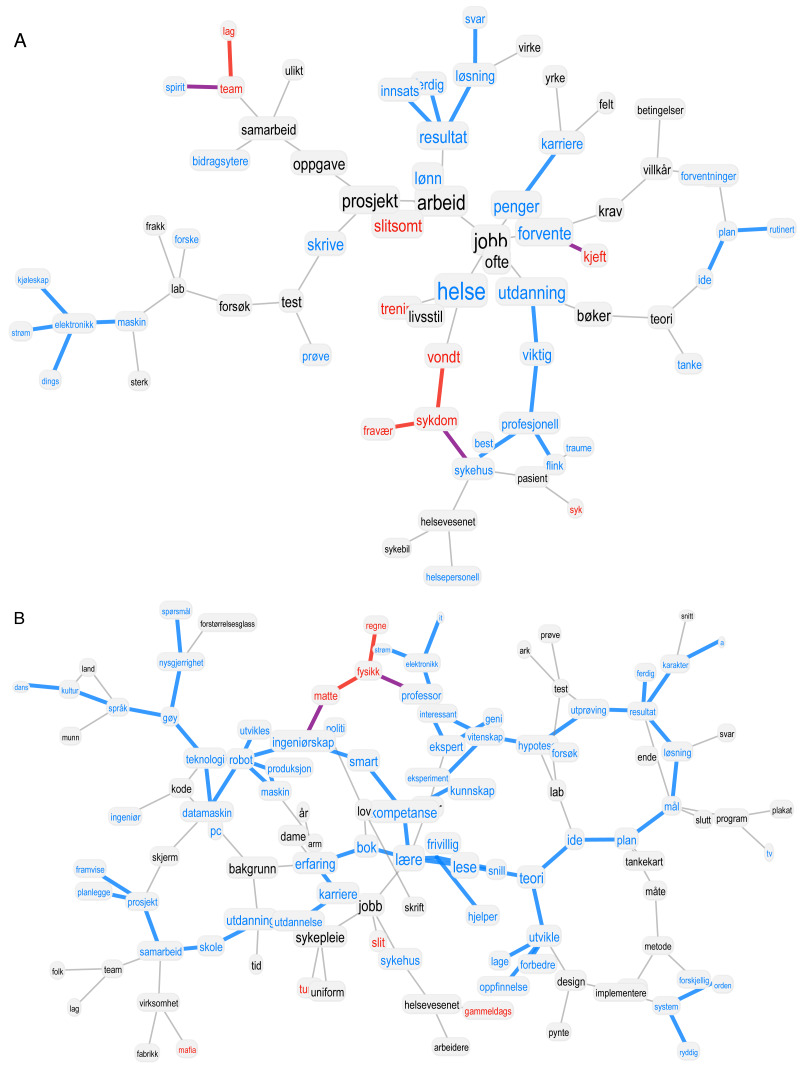
Visualisation of a forma mentis network. Largest connected component of forma mentis networks for Student 1 (nurse) before (A) and after (B) the summer job experience. Negative/positive/neutral words are highlighted in red, cyan and black, respectively. Links between positive (negative) words are highlighted in cyan (red). Purple links indicate associations between positive and negative concepts.

Figure 1 reports the largest connected components of the FMNs right at the start and at the end of the summer job experience for Student 1. This figure serves as an example for highlighting the key features of forma mentis networks: (i) conceptual associations between specific concepts and (ii) emotional labels (colour-coded for a better visualisation). Notice the prevalence of positive (cyan) concepts in both the networks, the presence of clusters of negative concepts only in the “before” network and the different layouts of associative knowledge structure before and after the summer job experience. Analogous changes are consistently present for all the considered students (see also the Supplementary Information), thus motivating additional inquiry of the microscopic changes of mindset structuring. Notice that the fine-grained structure of conceptual associations allows to reconstruct the stance of students on three levels at once, namely: (i) how students associated a given concept, (ii) how they perceived a given concept and (iii) how they linked a given concept to other positive/negative/neutral words. Stella and colleagues showed that the emotional surroundings of a given concept can reflect specific emotions, like negative concepts surrounded by other negative concepts eliciting stronger feelings of anxiety (Stella et al., 2019). We focus our longitudinal analysis on all these elements and traits of forma mentis networks.

**Figure 2:**
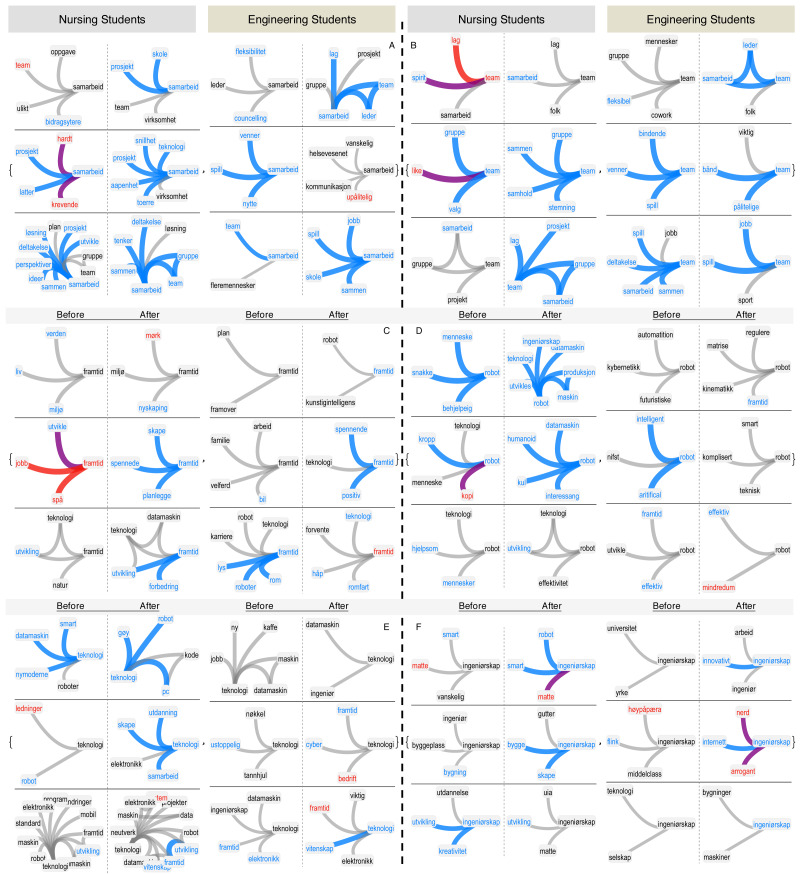
Longitudinal analysis of stances through FMNs. Neighbourhoods of “samarbeid” (collaboration, A), “team” (B), “framtid” (future, C), “robot” (D), “teknologi” (technology, E) and “ingenirskap” (engineering, F) in the forma mentis networks of students. In every subpanel, the nursing students’ forma mentis networks are on the left side while engineering students’ are on the right side. Negative/positive/neutral words are highlighted in red, cyan and black, respectively. Links between positive (negative) words are highlighted in cyan (red). Purple links indicate associations between positive and negative concepts.

Figure 2 reports the conceptual structure of concepts like “samarbeid” (collaboration, A), “team” (B), “framtid” (future, C), “robot” (D), “teknologi” (technology, E) and “ingenirskap” (engineering, F). Every row describes an individual FMN. A dashed line separates the pre- and post-intervention networks for the same person. For every concept, medical (engineering) students’ networks are reported on the left (right).

The most dramatic positive change in perception has been consistently registered for the word “team”. A positive improvement in perception is registered also in the related concept of “samarbeid” (collaboration). With the exception of one engineering student, all the others changed their perceptions of “collaboration” and “team” from neutral to positive and surrounded such concepts with more positive associates, i.e., with a *positive emotional aura* (cfr. Stella et al., 2019). This consistent change across most of the students is a strong quantitative indication that teamwork played an influential role in bringing the students together during the intervention. Also the composition of these positive emotional auras is concretely related to the summer job experience, with words important for collaboration such as “group”, “project” and “communication”. Other concepts of these auras are consistently related to a positive attitude towards others, like “openness”, “kindness” and “together”. These positive associations and auras indicate that the teamwork dynamics happening during the summer job was an enjoyable and rewarding experience. This quantitative finding, obtained and measured directly from the forma mentis networks, was also confirmed by the students in a feedback session after the data gathering (see also Text S2 for more details).

The intervention revolved around the creation of a healthcare robot. Figure 2 reports concepts related to this topic such as “framtid” (future), “robot”, “ingenirskap” (engineering) and “teknologi” (technology). Almost all the students improved their positive attitude towards the future and associated it with technological and professional concepts such as “teknologi” (technology), “datamaskin” (computer), “robot”, “karriere” (career) and “jobb” (job) by providing more positive, concrete and skill-oriented associations. Interestingly, changes in the perception of robots were prevalent in the nursing students but almost absent in the engineering students. Two out of three nursing students, who are also practising, attributed an overwhelmingly positive aura to “robot” and were able to associate it to more specific concepts like “humanoid”, “production”, “development” and “engineering” after the intervention. In comparison to the more general associations (e.g., “robot” or “human”) available before, the nursing students acquired a more detailed knowledge about the creation and development of a robot. These results indicate that the intervention made the future nursing professionals more aware about the benefits and features of robots. A similar pattern of improved and more concrete perceptions in the nursing students holds also for “technology” and “engineering”, which were related to “innovation”, “future” and “development” after the intervention. These findings indicate the development of positive professional relationships between robots, technology, innovation and professionals, which is going to become of primary importance in the Norwegian healthcare sector after the current investments in e-health (Rognstad, 2002; Zaytseva, 2016).

**Figure 3:**
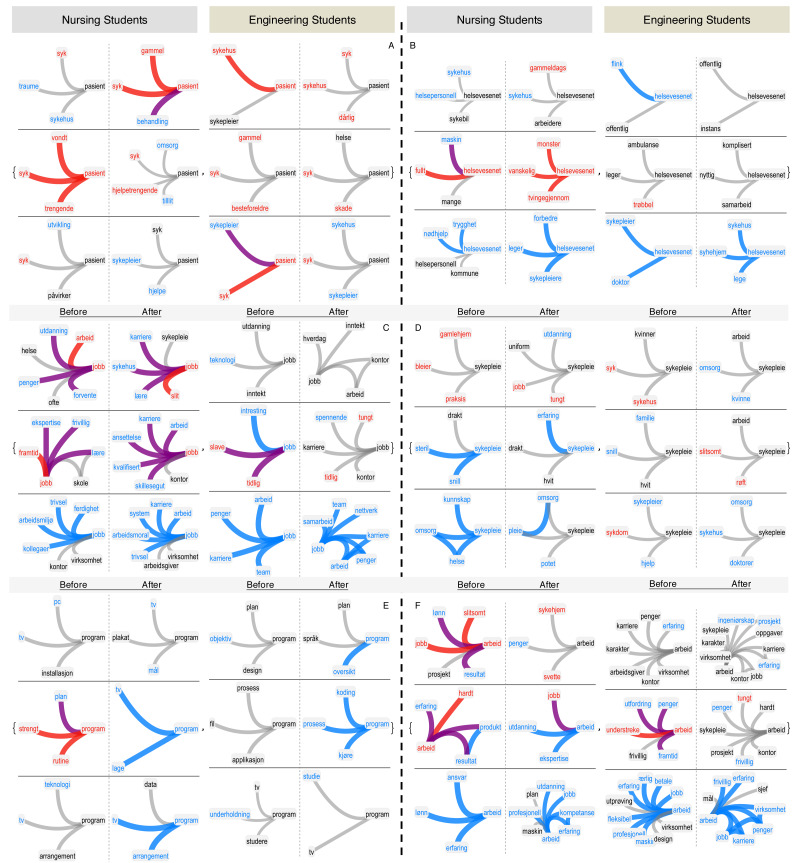
Longitudinal analysis of stances through FMNs. Neighbourhoods of “pasient” (patient, A), “helsevesenet” (healthcare sector, B), “jobb” (job, C), “sykepleie” (nursing, D), “program” (E) and “arbeid” (work, F) in the forma mentis networks of students. In every subpanel, the nursing students’ forma mentis networks are on the left side while engineering students’ are on the right side. Negative/positive/neutral words are highlighted in red, cyan and black, respectively. Links between positive (negative) words are highlighted in cyan (red). Purple links indicate associations between positive and negative concepts.

Nursing students improved their perception of engineering and robotics after the intervention. In other words, nursing students provided more positive, concrete and domain-specific associations towards engineering and robotics at the end of the summer job experience. Instead, engineering students did not improve their perception of the healthcare sector. As reported in Fig. 3, almost all the students perceived “helsevesenet” (healthcare sector) and “pasient” (patient) as neutral or negative concepts, associated mainly with other negative concepts like “syk” (sick), “vanskelig” (complicated) or “trengende” (needy). These associations indicate that both the nursing and engineering students perceived the health sector as a complicated system, and the intervention did not alter their perception. This is understandable, given the focus of the event on the engineering aspects of robotics for e-health. A further look at the concepts of “sykepleie” (nursing), “jobb” (job) and “arbeid” (work) confirms a conflicting perception students had about the nursing profession and its negative downsides. Interestingly, most of the nursing students, who are practising, perceived “job” as a negative concept but surrounded it with positive words related to “career”, “stipend” and “learning”, indicating the perceived economic relevance of having an occupation without highlighting any specific human aspect of nursing. This positive aura attributed to a negative perception of “job” and “arbeid” (work) might reflect an overall dissatisfaction with the human challenges posed by nursing in terms of dealing with patients and treating diseases. This is confirmed by the overall neutral perception of “nurse”, which is consistently associated with negative concepts like “sykdom” (sickness) and “tungt” (heavy). These results might be the reflection of a generally mixed perception of the nursing profession in Norway, as confirmed by other independent studies, cfr. (Rognstad, 2002). Given that the intervention, considered here, focused on the realisation of a e-health robot, it is expected for the students not to alter significantly their perception of the healthcare system and its nursing profession. Nonetheless, considering the crucial relevance that nursing has for healthcare (Rognstad (2002) and Atwal & Caldwell (2006); Begat, Ellefsen & Severinsson (2005)), forma mentis networks highlight the need for focused actions aimed at improving the general perception of the main rewards offered by the nursing profession by including more general and domain-specific associations.

Figure 3 reports also the perception that the nursing and engineering students had about “program”. While nursing students consistently associated it to “tv”, more technical associations were reported by the engineering students and were aimed at “coding”, “process” and “study”. These associations reflect a gap in the technical expertise between the nursing and engineering students. Despite this difference in backgrounds, it is remarkable that all the students were able to improve their perception of “robots” and “technology” (cfr. Fig. 2) and associate it to concrete aspects of engineering, computers and robotics. All the above results indicate a *peer-learning dynamics* Topping (2005) happening during the intervention, in which the nursing students were heavily influenced by the technical expertise of the engineering students and managed to improve their own perception and knowledge structure of robotics, engineering and innovation.

## Global Network Structure Identifies Core Concepts

Individual forma mentis networks identify changes that were shared by some students. How did these changes in stance translate on the whole group level? Let us focus on the collective forma mentis network obtained by merging together all the associations provided by students, as explained in the Methods section. Let us underline that global forma mentis networks consider associations that might be available to one student but missing to others and provide affect patterns based on the consensus of the whole group of individuals. These two elements make global forma mentis networks substantially different from individual networks.

Table 1 summarises different network metrics for the global forma mentis networks pre- and post-intervention. For the definition of mean local clustering, assortativity coefficient, diameter and mean path length we refer to the caption of Table 1 and to Newman (2018). The global forma mentis networks pre- and post-intervention are fully connected, include almost 700 concepts and a thousand of conceptual associations. These networks display a clustering coefficient one order of magnitude larger than random expectation (i.e., random networks preserving node and edge counts but randomising conceptual associations, also called configuration models (Newman 2018). This indicates a tendency for concepts to cluster between themselves, i.e., associates of a concept tend to share conceptual associations between themselves too. Compared to random null models, the empirical forma mentis networks also display a disassortative mixing by degree, so that nodes with few associations tend to link mainly to nodes with many connections. This reflects the construction of these networks, where cues received more connections than target words by construction. Empirical networks are also more tighly connected, with a smaller diameter and average path length, than random null models. Figure S7 reports the degree distributions of both these networks, which are found to be heavy-tailed, i.e., featuring highly connected nodes/hubs. All these elements indicate that the global forma mentis networks reported here display a small-world structure, linking tightly together in 4 or 5 *degrees of separation* hundreds of different concepts. This structure does not change between and after the intervention and is in agreement with previous findings linking small-worldness to universality features of both language and its cognitive counterpart, the mental lexicon (cfr. Siew et al., 2019). These results indicate that forma mentis networks share similar features to other networked representations of the mental lexicon (De Deyne, Navarro & Storms, 2013; Stella, Beckage & Brede, 2017; Vitevitch, Siew & Castro, 2018; Kenett, 2019; Sizemore et al., 2018; Siew et al., 2019; De Deyne et al., 2019).

**Table 1:** Table of network metrics for the global pre- and post-intervention FMNs and for the largest connected component of the core FMN (cfr. Methods). Clustering identifies how neighbours of a given concept are connected with each other. The assortativity coefficient identifies how poorly connected nodes tend to link to highly connected concepts. The diameter identifies the largest number of associations/links connecting any two concepts. Mean path length is the average number of associations/links connecting any two nodes. Rewired networks preserve the number of nodes and links of real networks but randomise all conceptual associations (i.e., configuration models). The error margins represent standard errors and are based on 100 randomised networks.

**Network metric**	**Before**	**After**	**Core**	**Before RW**	**After RW**	**Core RW**
Number of nodes	733	729	196	733	729	196
Number of links	1,075	1,090	244	1,075	1,090	244
Mean clustering	0.046	0.031	0.048	0.003 ± 0.001	0.003 ± 0.001	0.002 ± 0.001
Assortativity coeff.	−0.63	−0.61	−0.11	0.02 ± 0.01	0.02 ± 0.01	0.02 ± 0.01
Diameter	8	9	20	15	14	5
Mean path length	4.6	4.6	7.0	6.1 ± 0.3	6.2 ± 0.3	2.7 ± 0.2
Number of pos. words	279	327	72	–	–	–
Number of neu. words	347	305	108	–	–	–
Number of neg. words	116	97	16	–	–	–

Table 1 also reports a modest increase in the number of positive concepts between the pre- and post-intervention networks. Forma mentis networks are consistently made mostly of neutral and positive concepts, which indicate an overall mixed neutral/positive attitude towards the innovation, professional and job-specific concepts used as cues. Both the pre- and post-intervention networks report a tendency for concepts with the same emotional label to be connected to each other. At a significance level of 0.05, a Kendall Tau correlation of the labels of linked concepts is found both before (*τ*_*B*_ = 0.263, *N* = 1,075, *p* < 10^−6^) and after (*τ*_*A*_ = 0.297, *N* = 1,090, *p* < 10^−6^) the event. These correlations vanish in randomised networks preserving the same number of positive/negative/neutral concepts but with random conceptual associations (*τ*_*RA*_ = 0.027, *N* = 1075, *p* = 0.354; *τ*_*RB*_ = 0.028, *N* = 1,090, *p* = 0.335). These quantitative results indicate a tendency for students to associate concepts of similar emotional perception together, a phenomenon known as *emotional homophily* and found also in Stella et al. (2019).

**Figure 4:**
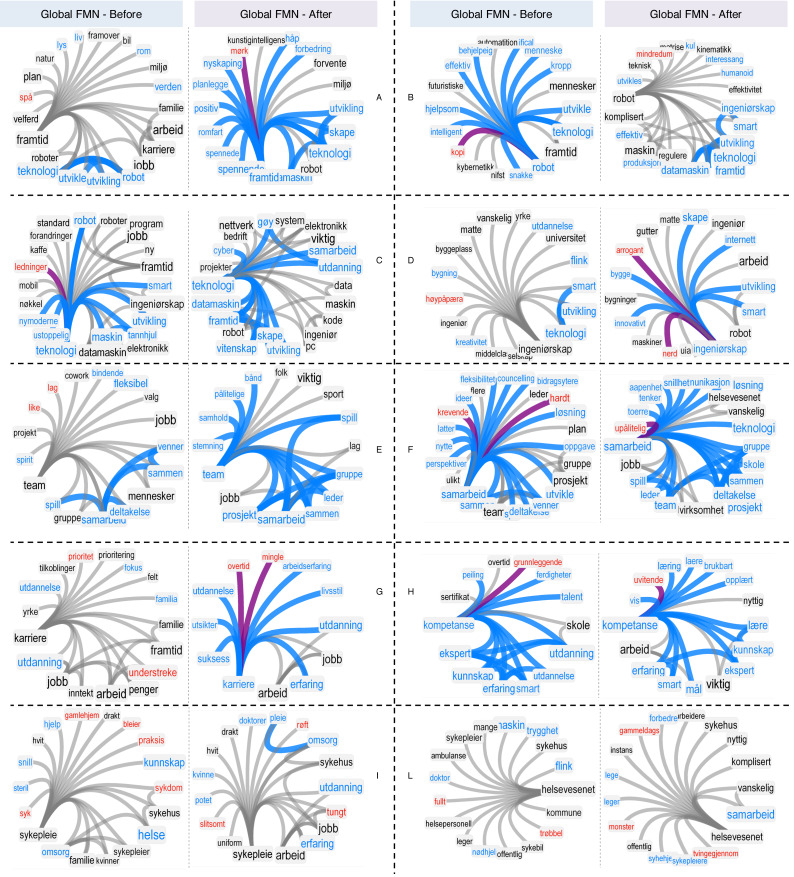
Global forma mentis networks encode group-level stances before and after the intervention. From the “before” and “after” global forma mentis networks of students, local neighbourhoods of: “framtid” (future, A), “robot” (B), “teknologi” (technology, C), “ingeniørskap” (engineering, D), “team” (centre, E), “samarbeid” (collaboration, F), “karriere” (career, G), “kompetanse” (compentence, H), “sykepleie” (nursing, I) and “helsevesenet” (L). In every subpanel nursing and engineering students’ are aggregated together. Negative/positive/neutral words are highlighted in red, cyan and black, respectively. Links between positive (negative) words are highlighted in cyan (red). Purple links indicate associations between positive and negative concepts. Concepts of higher closeness centrality appear with a larger font.

The above global correlations do not characterise the specific stance of the group of students, which can be achieved by a microscopic comparison of neighbourhoods, instead. Figure 4 reports how different concepts were associated at the group level before and after the event. The most dramatic improvements are relative to “team” and “karriere” (career), which passed from a neutral perception and aura to a positive perception and aura. After the intervention, the whole group of students associated team with “viktig” (important), an association that had not been reported by any of them before. A similar association appeared also on “kompetanse” (competence). These results represent an additional finding about the important role played by teamwork during the summer job experience, which is confirmed also by the constantly positive perception and aura of “samarbeid” (collaboration) at group level (cfr. Figure 4). Also the local clustering of “team” improved, with concepts like “leader”, “group”, “together”, “job” and “project” becoming more tightly interconnected than in the pre-intervention network. This indicates the happening of a positive, leader-oriented group dynamics during the event. Another positive change was registered also for the “framtid” (future), which was consistently associated with “teknologi” (technology) before and after the intervention. Additionally, “technology” itself remained a positively perceived concept. These results indicate that the *whole* group of students already had a positive attitude towards “innovation”, “technology” and “future” even before the intervention started. This is indeed expected, as only students with a genuine passion for innovation were involved in the summer job. The group also preserved this positive stance throughout the whole experience and displayed a more positive attitude towards “engineering”, as reflected also in the individual formal mentis networks. Interestingly, concepts related to healthcare and nursing remained neutral also at group level. The constellation of positive, neutral and negative concepts surrounding both “sykepleie” (nursing) and “helsevesenet” (healthcare sector) summarise the complex beliefs about the pros and cons of professional healthcare as perceived by the young nursing and engineering professionals.

Valence is not the only difference between individual and global forma mentis networks. Notice that the combination of conceptual associations from the different individual mindsets can greatly change the general knowledge available to the whole group, i.e., an individual might provide conceptual links bridging together concepts that would otherwise be far apart. This factor makes the analysis of the group forma mentis network at the global scale quite different compared to the previous longitudinal analysis. We use closeness centrality in order to evaluate potential changes in the global forma mentis networks before and after the event. Closeness indicates on average the minimum number of conceptual associations linking one concept to all other ones in a connected component of a network and has been successfully used for detecting central words for language acquisition and use (see Methods). Also, notice that a comparison between the “before” and “after” forma mentis networks is appropriate here, as both these networks are fully connected and have analogous size and connectivity (cfr. Table 1).

Table 2 reports the most extreme changes in closeness centrality for relevant concepts in the FMNs before and after the event. The drastic increase of centrality for “ingenir” (engineer) reflects the engineering context of the summer job experience, and it was caused by the availability of new connections with “design”, “code” and “technology”, originating from both the nursing and engineering students (see Fig. 4). This quantitative finding indicates the acquisition of an increased awareness of the professional aspects of engineering in the whole group of students during the intervention. The increase in closeness centrality/conceptual relevance of other professional concepts, such as “matematikk” (mathematics) and “kode” (code), further confirms the acquisition and restructuring of knowledge around relevant aspects of the event, revolving around the creation of an e-health robot. Also, concepts like “sykepleie” (nursing) and “sykehus” (hospital) became more central after the intervention, indicating an increased awareness of students about the structure and relevance of the healthcare sector. Last but not least, the increase in centrality of “suksess” (success) and its positive perception both indicate how positively the whole group of students perceived the summer job.

**Table 2:** Table of ranks based on closeness centrality (Clos.) of words in the global forma mentis networks obtained before and after the event. The reported concepts are those with the largest excursions and in the top 10-th percentile of the distribution of closeness centrality in the post-intervention global FMN.

**Concept**	**Clos. Rank (Before)**	**Clos. Rank (After)**	**Difference**
ingeniør (engineer)	374	46	+329
matematikk (maths)	198	34	+164
kode (code)	163	56	+107
suksess (success)	142	55	+107
skape (create)	108	20	+88
nettverk (network)	90	12	+78
sykepleie (nursing)	89	53	+36
sykehus (hospital)	54	19	+35
samarbeid (collaboration)	18	6	+12

## Core Link Comparisons Highlight Robust Features of Mindsets

The global comparison focused mostly on differences between the pre- and post-intervention FMNs. Here we focus on those conceptual associations that were present already at the beginning of the event and persisted until its end in the global mindset of students. We name these as “core” associations and, as mentioned above, we interpret them as the backbone of forma mentis networks, indicating the strongest links between concepts. Notice that we identify a “backbone” that is not formed by filtering links according to node similarity or network optimisation processes, as reported in Foti, Hughes & Rockmore (2011). Our backbone rather identifies strong conceptual associations that persisted over time. The analysis of the resulting core forma mentis network can be informative about the conceptual knowledge representing the group of students that participated in the event.

The core FMN included a largest connected component with 196 concepts and 244 associations. Figure S1 reports the degree distribution of the core network. Given the low connectivity and size of the network, adopting only one measure of network centrality might produce biased results (Newman, 2018). We resort here to a combination of closeness and graph distance entropy, which was successfully used in small-sized networks for identifying words of relevance for cognitive development in a learning environment (Stella & De Domenico, 2018). Closeness identifies on average how far a concept is from all others but it does not provide info on how consistently far or close a concept is from its connected neighbours. Graph distance entropy ranges between 0 and 1 and it is lower for nodes that have more short-length connections to all other nodes in a given connected component. Hence, graph distance entropy can be considered as a measure for direct accessibility and centrality of nodes, independently of network size (Stella & De Domenico, 2018). For more details, we refer to the Methods.

**Figure 5:**
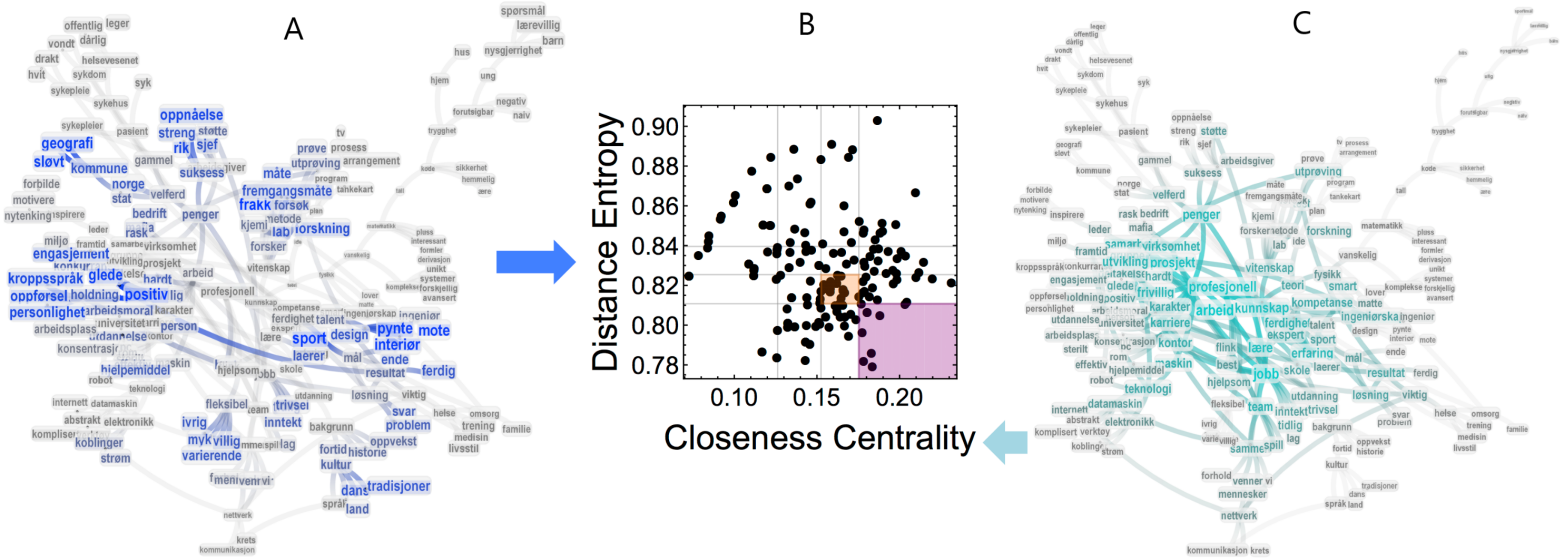
Distance entropy cartography of the core FMN reveals characterising features of the students’ mindset. Graph distance entropy (A) and closeness centrality (C) become coordinates for a network cartography of relevant nodes within the core forma mentis network (B). The graph distance cartography reported in the centre is a 2D map where concepts/nodes are plotted according to their coordinates. Concepts of relevance are identified as being in both the upper and mid-upper quartiles of the closeness and entropy distributions (see purple and orange areas in (B), respectively). Notice that lower distance entropy identifies more central nodes. In (A), nodes with a higher graph distance entropy are larger and highlighted in blue. In (C), nodes with a higher closeness centrality are larger and highlighted in green. Entropy and closeness identify different categories of concepts.

Figure 5 highlights the most central concepts in the largest connected component of the global core forma mentis network in terms of graph distance entropy (left) and closeness centrality (right). Table 3 reports the top-five cues and target words with the highest closeness and graph distance entropy. We have to separate ranks for cues and targets. Although not all cues are the same, they differ in comparison to target words. In fact, cues appear in the networks of every individual by construction while target words do not. The above network measures report different categories of concepts. Closeness highlights mainly cues related to the professional sphere (arbeid/work, jobb/job, profesjonell/professional, prosjekt/project and lære/learn) while graph distance entropy captures mainly cues related to the intervention aims (kommune/municipality, lab, design, holdning/attitude and suksess/success). Among targets, closeness highlighted mainly concepts related to personal development and growth (kunnskap/knowledge, penger/money, utvikling/development, hardt/hard and smart). Distance entropy identified targets without a clear relationship (sport, positiv/positive, frakk/coat, interir/interior and mte/style). Notice that all the words in the network are far from being star centres, i.e., they have a distance entropy far higher than 0, see Fig. 5. Hence, having a lower distance entropy might only highlight concepts that are consistently far from all other concepts.

**Table 3:** Table of top-5 ranks based on closeness centrality and graph distance entropy for cues and targets within the core FMN.

**Closeness (Cues)**	**Closeness (Targets)**	**Dist. Entropy (Cues)**	**Dist. Entropy (Targets)**
arbeid (work)	kunnskap (knowledge)	kommune (municipality)	sport
jobb (job)	penger (money)	lab	positiv (positive)
profesjonell (professional)	utvikling (development)	design	frakk (coat)
prosjekt (project)	hardt (hard)	holdning (attitude)	interiør (interior)
lære (learn)	smart	suksess (success)	måte (style)

In order to highlight *central* concepts that are also at the same distance from all other nodes, it is necessary to combine closeness centrality and graph distance entropy through a network cartography (see Fig. ??). To this aim, we considered concepts in the upper quartile of the distribution of closeness centralities and in the lower quartile of graph distance entropies. This cartography highlighted the following concepts: “hard”, “income”, “early”, “well-being”, “pleasure”, “positive”, “sport”, “research”. Such concepts are central in the core forma mentis network, which represents the most robust conceptual associations characterising the mindset of the students participating in the summer job experience. These concepts express a mindset revolving around research, personal well-being and professional aims. The other relevant concepts, as obtained from the mid-upper quartiles, also relate to an interest for science, well-being and professional growth: “relationship”, “people”, “friends”, “we”, “team”, “old”, “education”, “workplace”, “concentration”, “pc”, “room”, “sterile”, “effective”, “talent”, “engineer”, “method”, “scientist”, “chemistry”.

Importantly, graph distance cartography indicated that in the students’ global mindset, positive relationships with the others and team-play are fundamental, further corroborating the previous findings about the relevance of collaboration, reported in this work, by means of a different, entropy-based approach Stella & De Domenico (2018).

## Discussion

Forma mentis networks represent how individuals structure and evaluate their knowledge and perception of a given topic in the form of a complex network of conceptual “free” associations (De Deyne, Navarro & Storms, 2013) (e.g., “work” and “career” remind of each other) and valence labels (Kuperman et al., 2014) (e.g., “work” is perceived as a “positive” concept), cfr. (Stella et al., 2019). This cognitive representation builds upon extensive research in psycholinguistics and cognitive network science (Aitchison, 2012; De Deyne, Navarro & Storms, 2013; Kuperman et al., 2014; Kenett et al., 2017; Kenett, 2019; Siew et al., 2019) and, in comparison to other powerful machine learning approaches (Mohammad, Sobhani & Kiritchenko, 2017; Mohammad et al., 2016), it has the main advantage of enabling access to the microscopic layouts of affect and conceptual associations.

Here we used forma mentis networks in order to highlight changes and similarities in the mindsets of a small group of nursing and engineering students who took part in a summer job/project focusing on assisted healthcare with robotics. The analysis of such population is of relevance when related to the challenge of silo-driven education in Norway (Thistlethwaite, 2012; Zaytseva, 2016), where professionals are trained only in specific backgrounds, e.g., nursing students are trained only in nursing while engineering students only in engineering. As a result, the new generation of workers in the labour market can work within only one domain of practice, which makes it very hard for trainees to adapt to the introduction of new methods of work and tools provided by emerging technologies. This makes collaboration with technology-focused silos impossible and minimises chances of domestic Norwegian innovation in the areas across healthcare and engineering (Zaytseva, 2016). By applying the framework of forma mentis networks to an e-health professional training, we aimed at demonstrating the occurrence of changes in the mindsets of young students, representing the silo-driven study programmes, happening when people with different backgrounds interact and collaborate in one holistic socio-technical process.

To this aim, we focused our stance detection around, but not limited to, 75 cues (see Supplementary Information) related to innovation, professional growth and technical expertise from engineering and healthcare. Particular relevance was given to detecting improvements in these mindsets, mainly in terms of structuring stances towards e-health topics by providing more positive, domain-specific, skill-oriented and concrete associations. The testing of the presence of emotional and knowledge-specific associations was enabled by the availability of microscopic conceptual links, which were analysed in detail in the current analysis. Despite the limiting small sample size of 6 individuals, only strong conceptual associations, i.e., those provided at least twice in the overall experiment, where analysed, in accordance to the best practice in other works based on free association networks (De Deyne, Navarro & Storms, 2013; Kenett et al., 2017).

A longitudinal analysis of the FMNs from individual students highlighted consistent and drastic changes in the stance towards “team” and “engineering”, which were strongly positively perceived at the end of the professional experience. In synergy with the overwhelmingly positive stance towards “collaboration”, this finding indicated the crucial relevance and the positive outcomes that communication and team play had in enabling a team of students with different backgrounds to achieve the completion of an engineering-oriented project. Our results are in agreement with other studies investigating performance of multidisciplinary teams in healthcare (Begat, Ellefsen & Severinsson, 2005; Atwal & Caldwell, 2006; Soukup et al., 2018). Atwal and colleagues (Atwal & Caldwell, 2006) reported how nurses perceived the lack of effective teamwork as the most daunting challenge in multidisciplinary working environments. Further, Begat and colleagues (Begat, Ellefsen & Severinsson, 2005) reported how a good communication correlated with lower loads of stress and anxiety. Notice that, whereas these approaches described this perspective of nurses on larger samples than ours, they also focused only on one point in time. Instead our FMNs and their cognitive interpretation of semantic knowledge provide whole temporal and learning dimensions, in the sense of Koponen, Kokkonen & Nousiainen (2017), Lydon-Staley et al. (2019), to the description of stances. Within these temporal/learning dimensions, we find that both nursing and engineering students restructured their mental lexicon/mindset/forma mentis around the same tested cues with the outcome of making concepts like “teamwork” and “collaboration” as more central and at shorter semantic distance from other concepts (as measured by closeness centrality). Students’ feedback indicated that the above network restructuring coincided with students’ self-reporting that the most appreciated aspect of the summer job event was the creation of a positive environment, promoting communication and collaborative teamwork (see Text S2).

Other differences among individual mindsets were identified and mainly related to different personal backgrounds between nursing and engineering students. Nursing students were found to have considerably improved their perception of robots at the end of the experience. This mindset change was absent in engineering students. The latter students were also capable of accessing associations related to coding expertise like “program” and “application” or “program” and “objective”, whereas nursing students consistently associated “program” to “tv” before and after the intervention. These patterns highlight differences in the specific expertise of individuals. Although the relationship between conceptual associations and domain specific expertise has not been fully explored yet (Aitchison, 2012), a few pioneering approaches have recently established how the structure of semantic networks can be predictive of students’ performance in quizzes (Siew, 2019; Tyumeneva, Kapuza & Vergeles, 2017). The further exploration of a link between network structure and domain expertise represents an interesting future research direction for forma mentis networks, also in terms of investigating the effects of shared knowledge and of prior-knowledge over learning outcomes in the sense of Koponen, Kokkonen & Nousiainen (2017).

The aggregation of individual associations and the consensus of affective labels led to global forma mentis networks. The latter described the semantic knowledge and average perception of students as a whole group. These networks highlighted a positive attitude towards “future”, “engineering” and “technology”, as expected from students who enrolled in an innovation summer job. Nonetheless, this professional experience had a beneficial influence in improving the students’ perception of professional career development. Concepts like “nursing” and “healthcare” were perceived as mixed, i.e., neutral and associated mainly with a constellation of other negative/neutral/positive concepts. These results are compatible with previous studies in the Norwegian healthcare system (Begat, Ellefsen & Severinsson, 2005) reporting nursing as a profession perceived as stress- and anxiety-inducing by nurses themselves. A previous study (Stella et al., 2019) highlighted anxiety patterns in student populations through forma mentis networks, but in here the small sample size makes it impossible to draw general conclusions about how a whole professional category perceives a profession. This clearly represents a limitation of the current analysis, which can be relieved with additional data gathering in future yearly editions of the summer job event. Instead, the above results represent evidence that forma mentis networks can be used for tracing the expansion of emerging competence networks (Zaytseva, 2016; Koponen, Kokkonen & Nousiainen, 2017) and the occurrence and outcomes of transdisciplinary collaborations across education domains.

Last but not least, the temporal snapshots of the pre- and post-intervention mindsets of students were used for building a “core” forma mentis network, including conceptual associations persisting over time. By combining semantic distance measures of relevance for cognitive network science (Kenett et al., 2017; Goldstein & Vitevitch, 2017; Stella & De Domenico, 2018; Stella, 2019), namely closeness (Newman, 2018) and graph distance entropy (Stella & De Domenico, 2018), this unsupervised approach, i.e., requiring no prior knowledge about students’ mindsets, identified the key concepts characterising the personal and professional profile of the group of selected students. Positive relationships towards others, a passion for career development and openness towards collaboration and communication were found to be the most relevant concepts in the networked mindsets of students’, further corroborating analogous evidence based on multiple different, specific survey-based approaches (Begat, Ellefsen & Severinsson, 2005; Atwal & Caldwell, 2006; Soukup et al., 2018). The qualitative feedback gathered from students confirmed the relevance of teamwork and professional interactions during the event (cfr. Text S2).

Notice that one advantage of the approach of FMNs is its generality and flexibility, as it can span several stances about different cognitive spheres at once without the strict need for deception filtering and the computation of statistical correlations that are essential in self-reporting surveys (for an example, see the factor analysis, rotation renormalisation and correlation thresholding in Visser-Wijnveen, Van der Rijst & Van Driel (2016)). Also the cognitive data behind forma mentis networks is somehow self-reported by individuals, but the lack of specifically correct or wrong answers and the need for quickly completing the task without mind-wandering both drastically reduce the occurrence of deception. Another advantage is that no correlations have to be computed for building snapshots of forma mentis networks, differently from survey-based models.

Indeed, FMNs suffer from several limitations, mainly related to the small network size and the need for considering multiple shapshots of networks or replicas of individual connections. In this case, we compensated the small sample size by focusing on repetitions of associative patterns either across individuals or consistently between pre- and post-intervention snaphots. Strong free associations are usually considered as having been produced by at least two different participants (De Deyne, Navarro & Storms, 2013; De Deyne, Navarro & Storms, 2013; De Deyne et al., 2019). This methodology enabled the identification of strong conceptual changes in the mindsets of even such a small group of students. Expanding the approach of FMNs to larger or different samples would be highly relevant to institutions providing innovative healthcare services. Several questions might be investigated *at once*. For instance, how does a given group of future nursing professionals structure their perception around the idea that ”the patient has to be in the centre of the service provision”? How do these trainees exactly feel about the profession they are about to perform? Further, how does a team of engineering professionals feel when placed in a collaborative context with healthcare workers, who know well how demanding the service provision for patients is but have no minimal knowledge about the challenges of developing technological tools? Our results indicate that forma mentis networks can generate knowledge of relevance for addressing these questions with the above advantages in comparison to standard surveys, at the cost of gathering cognitive data.

For future research we reserve the aim to further relate the structure of forma mentis networks to additional quantitative data. Potential candidates are recent network-based indicators about personality, such as creativity scores (Kenett, 2019; Stella & Kenett, 2019), openness to experience indicators and (Christensen et al., 2018) curiosity metrics (Lydon-Staley et al., 2019). Based on the availability of these additional datasets, whose collection requires time and effort, then changes in forma mentis networks could be described also in terms of persisting and evolving personality traits.

## Conclusions

Our current results with forma mentis networks provide quantitative ways for accessing and understanding the mindsets of individuals during professional growth. More in detail, this study showed how either persistent features or changes over time in the mindset of individuals or groups can be detected, described and reconstructed in detail through forma mentis networks. Microscopic access to the knowledge encapsulated within mental stances and beliefs of people opens novel exciting challenges for overcoming silo-driven educational approaches, not relevant for the job market, and building data-informed interventions and scenarios of effective professional training empowered by computational cognitive science.

##  Supplemental Information

10.7717/peerj-cs.255/supp-1 Supplemental Information 1 Dataset for the forma mentis networks of individual students before and after the intervention Every data file includes network links and valence attributes as reported in the main text.

10.7717/peerj-cs.255/supp-2 Supplemental Information 2 Supplementary Information

## Additional Information and Declarations

### Data Availability

The following information was supplied regarding data availability: Data is available at OSF: Stella, Massimo. 2019. “Supplemental Materials for: Forma Mentis Networks Map How Nursing and Engineering Students Enhance Their Mindsets about Innovation and Health during Professional Growth.” OSF. October 8. osf.io/jmbzp.
