# Supplementary figures and images for: Forma mentis networks map how nursing and engineering students enhance their mindsets about innovation and health during professional growth
Source: PeerJ Comput Sci. 2020 Mar 2;6:e255. doi: 10.7717/peerj-cs.255 (PMC7924483; doi:10.7717/peerj-cs.255)

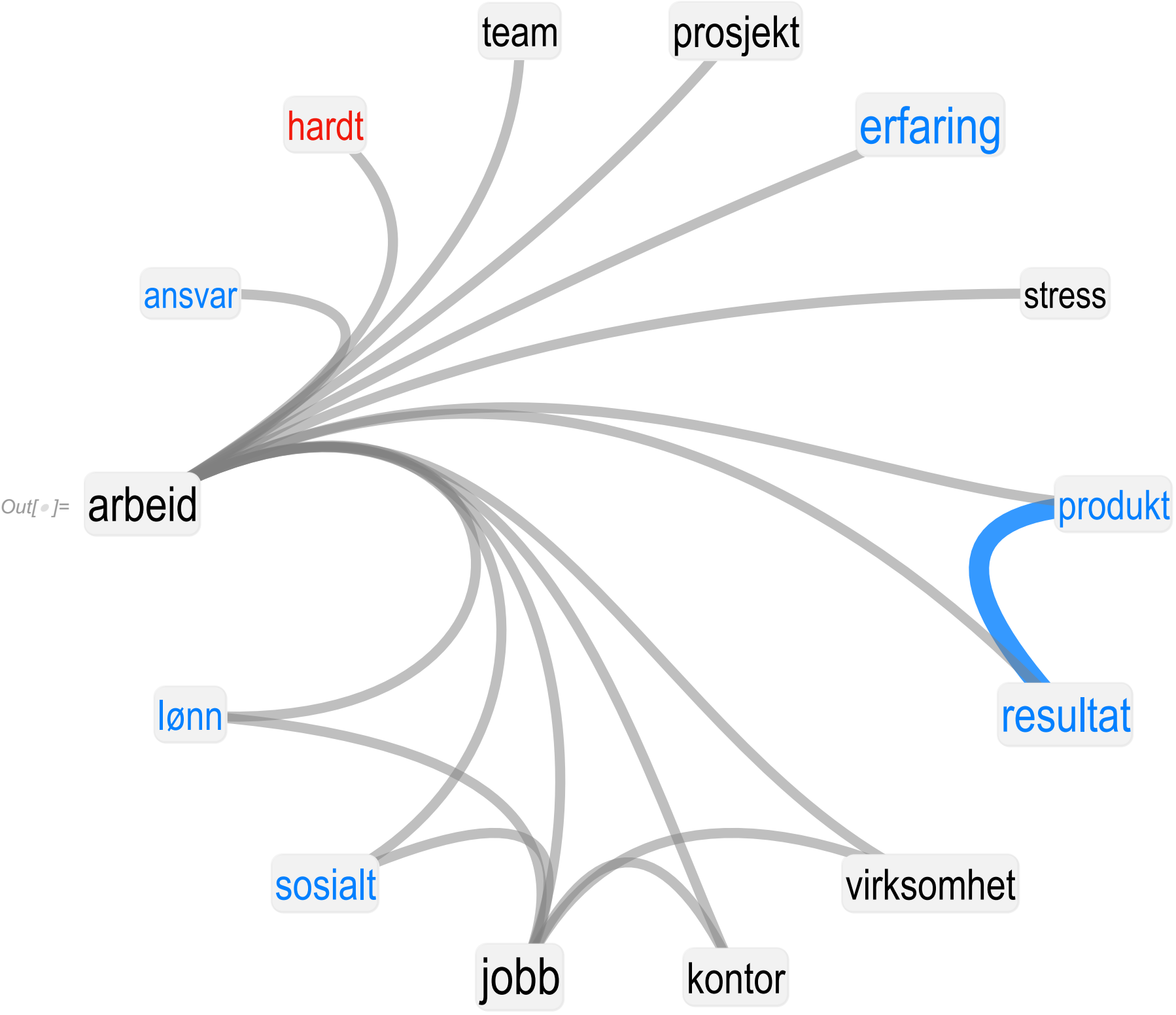

Supplement: Supplemental Information 1 — Every data file includes network links and valence attributes as reported in the main text. [file peerj-cs-06-255-s001.zip › Arbeid Females.pdf]

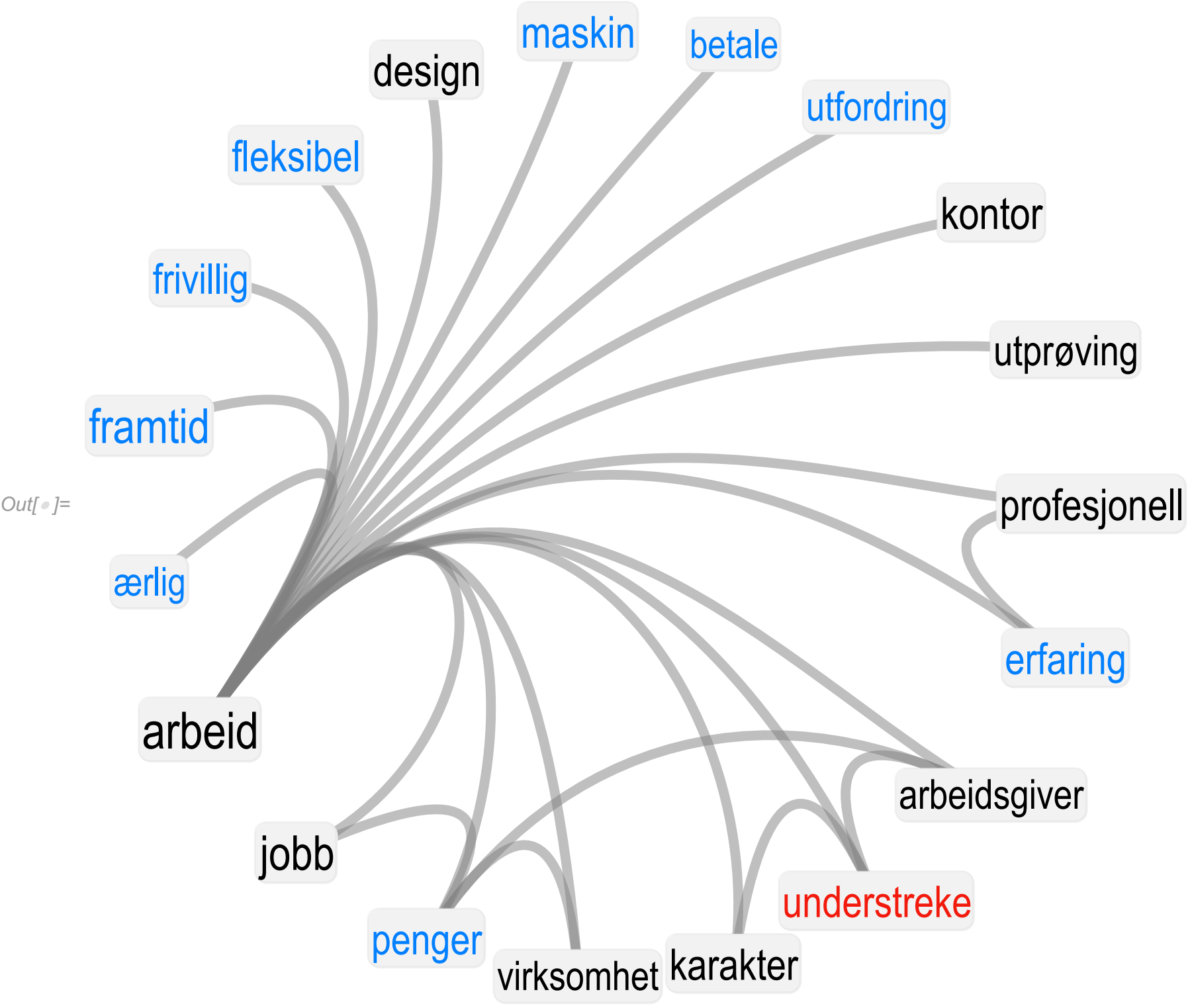

Supplement: Supplemental Information 1 — Every data file includes network links and valence attributes as reported in the main text. [file peerj-cs-06-255-s001.zip › Arbeid Males.pdf]

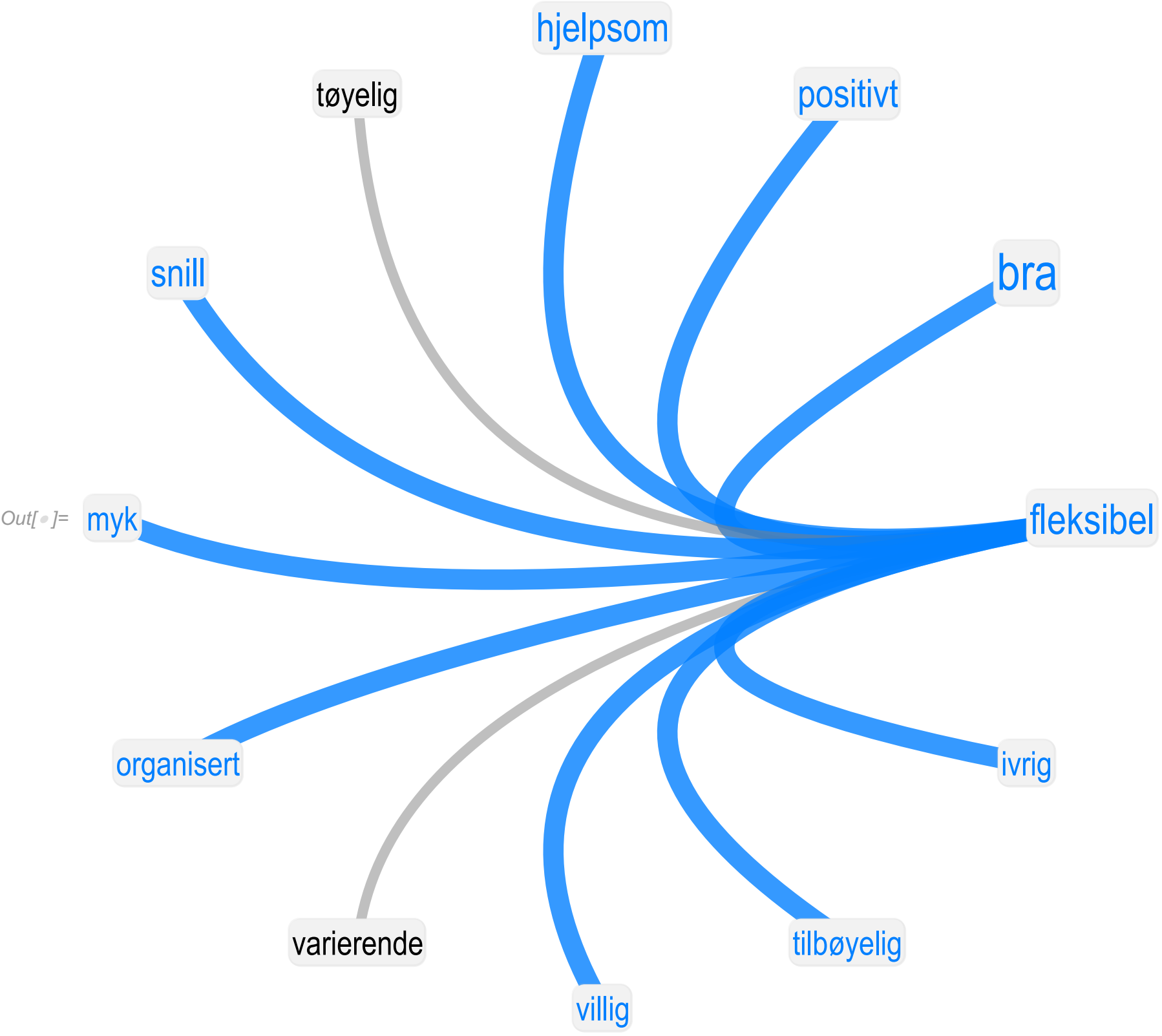

Supplement: Supplemental Information 1 — Every data file includes network links and valence attributes as reported in the main text. [file peerj-cs-06-255-s001.zip › Fleksible Females.pdf]

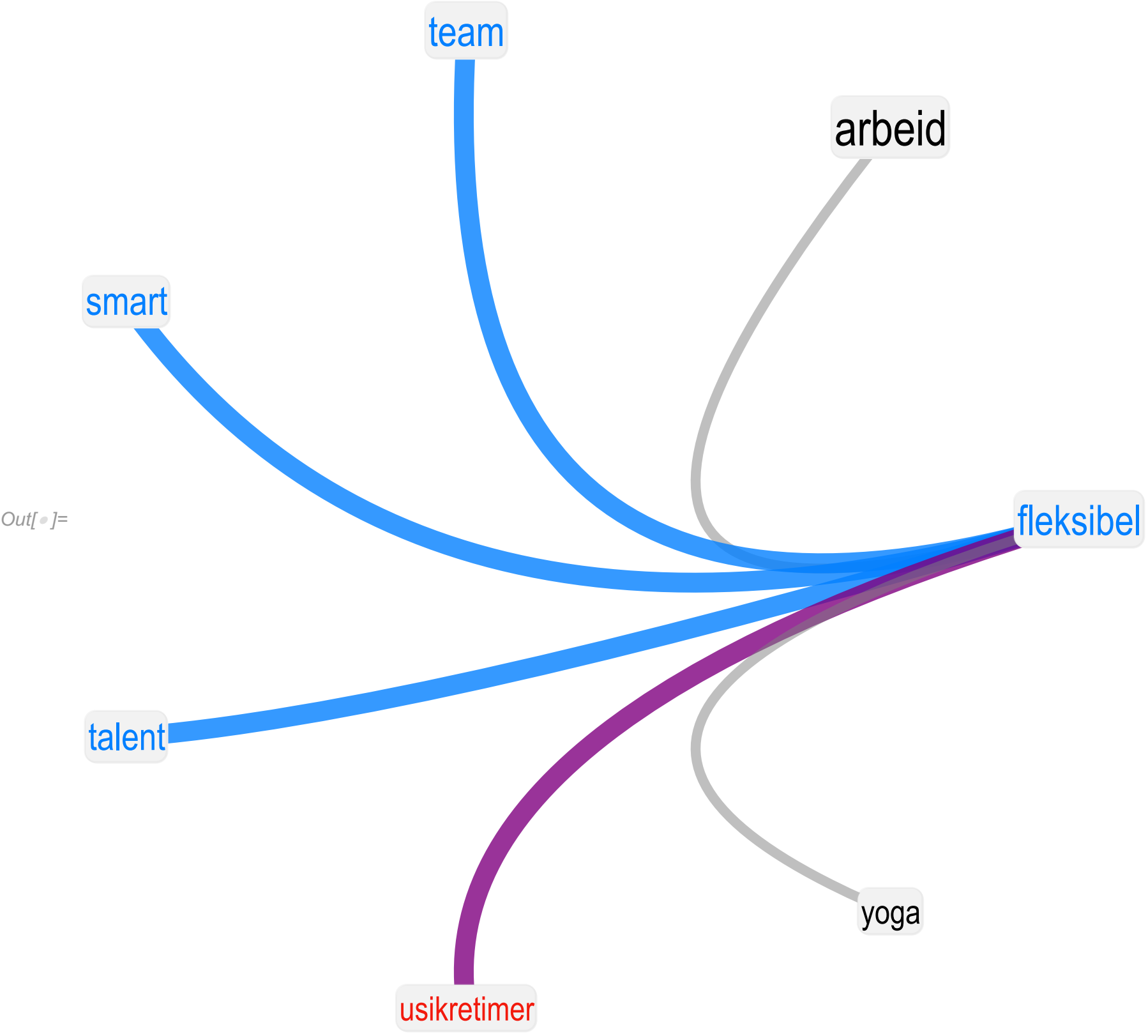

Out[•]=

Supplement: Supplemental Information 1 — Every data file includes network links and valence attributes as reported in the main text. [file peerj-cs-06-255-s001.zip › Fleksible Males.pdf]

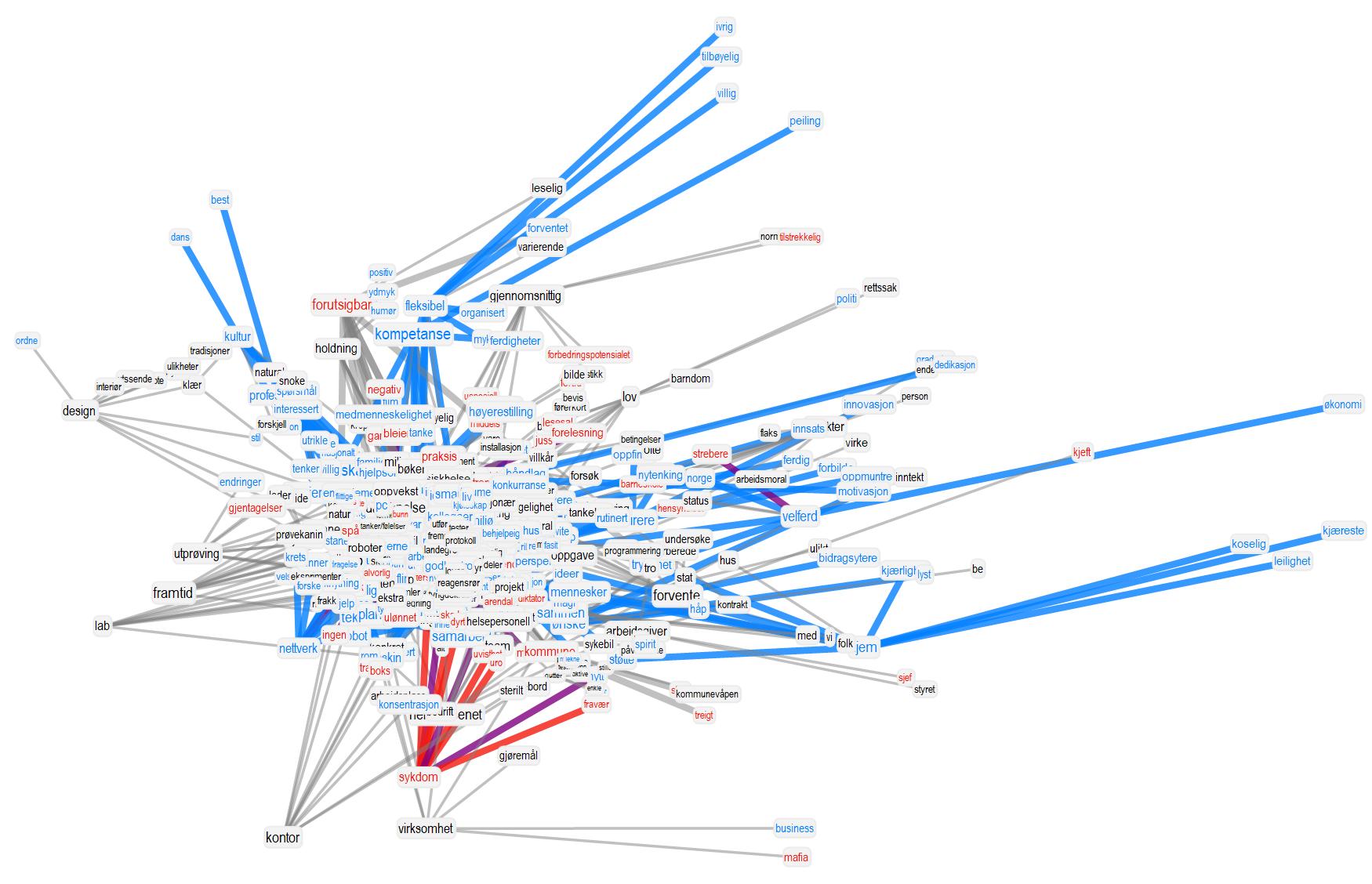

Supplement: Supplemental Information 1 — Every data file includes network links and valence attributes as reported in the main text. [file peerj-cs-06-255-s001.zip › FM All Females - Spaghetti.jpg]

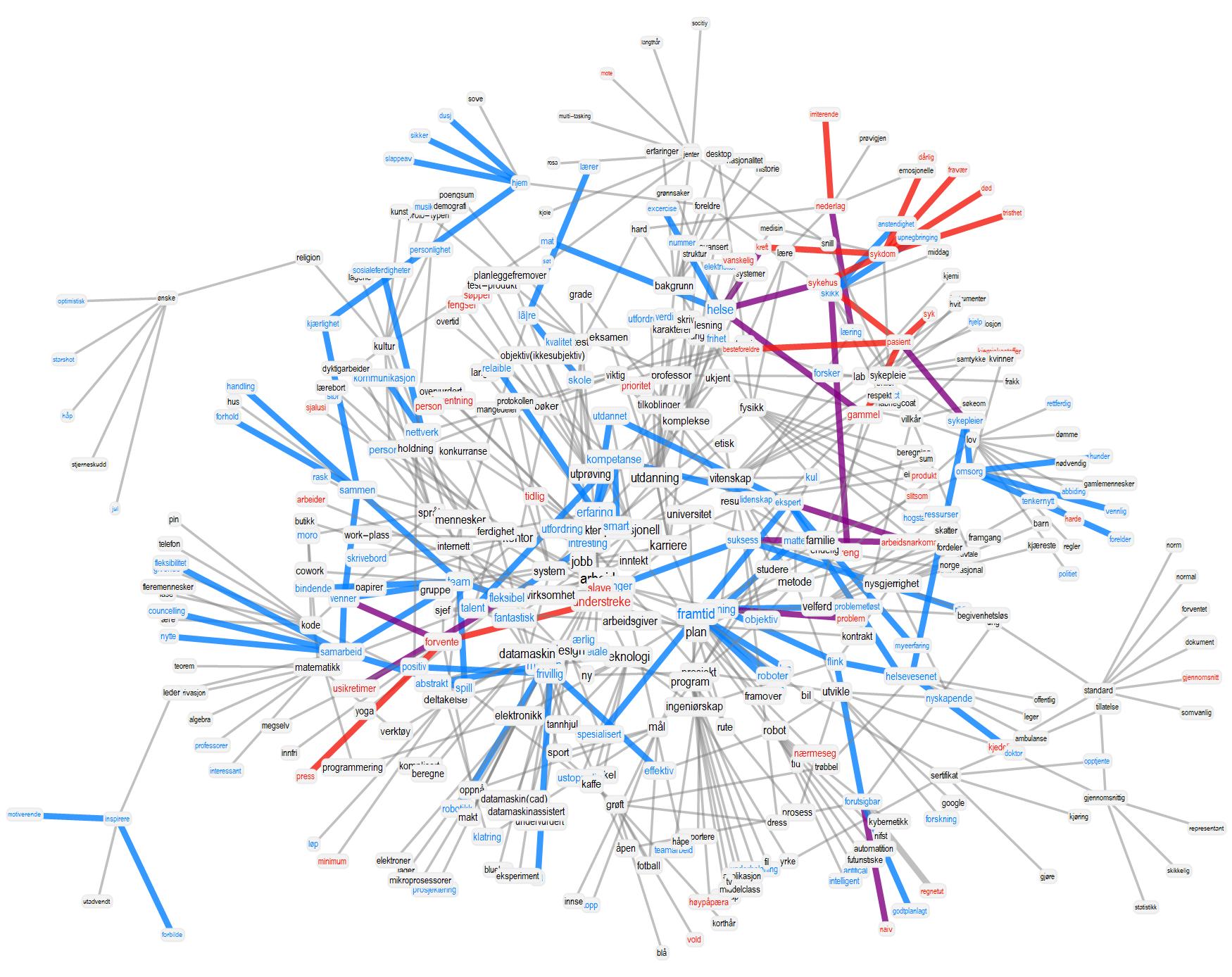

Supplement: Supplemental Information 1 — Every data file includes network links and valence attributes as reported in the main text. [file peerj-cs-06-255-s001.zip › FM All Males - Spaghetti.jpg]

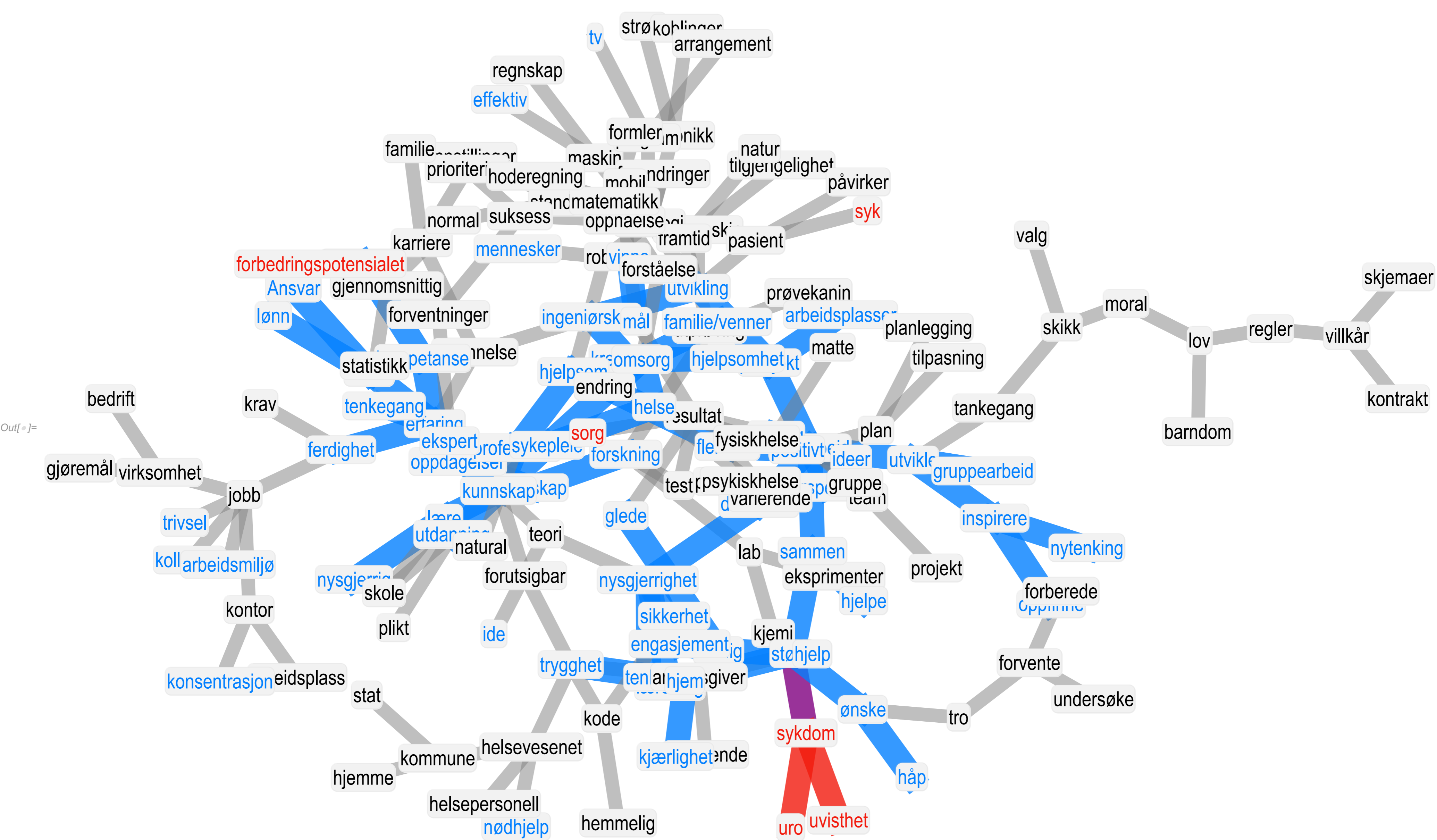

Supplement: Supplemental Information 1 — Every data file includes network links and valence attributes as reported in the main text. [file peerj-cs-06-255-s001.zip › FM Benedykte.pdf]

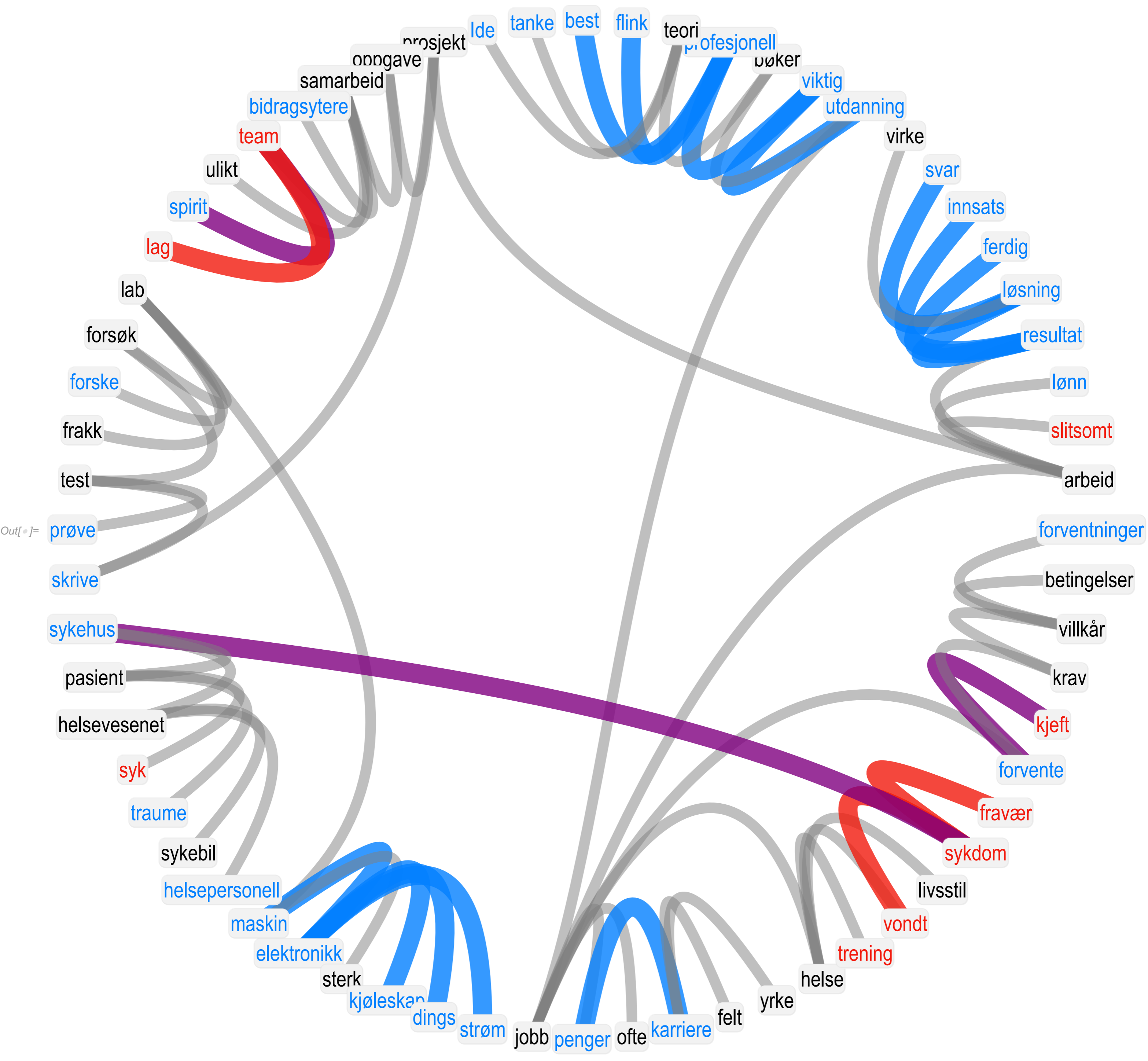

Supplement: Supplemental Information 1 — Every data file includes network links and valence attributes as reported in the main text. [file peerj-cs-06-255-s001.zip › FM Cornelia.pdf]

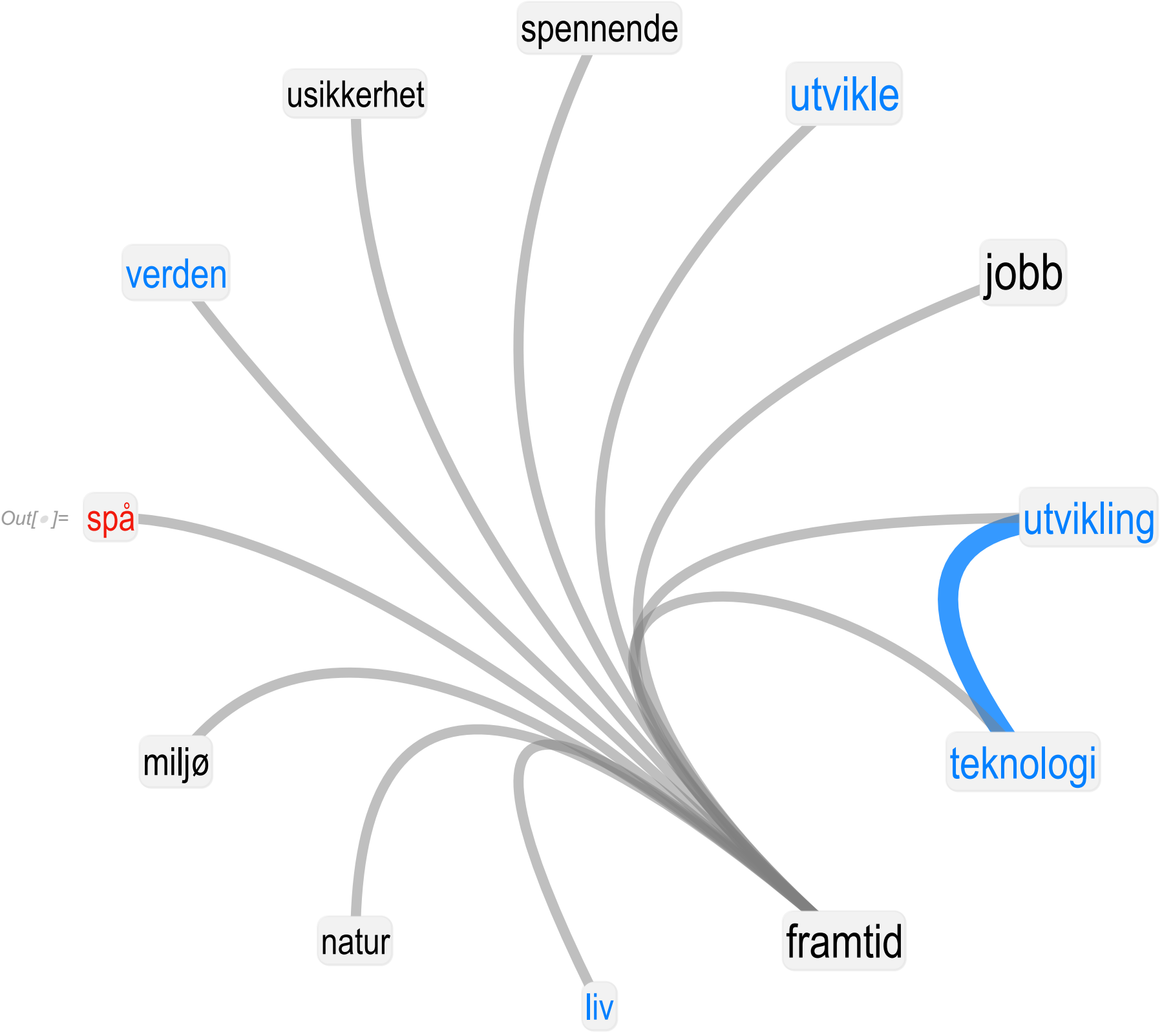

Supplement: Supplemental Information 1 — Every data file includes network links and valence attributes as reported in the main text. [file peerj-cs-06-255-s001.zip › Framtid Females.pdf]

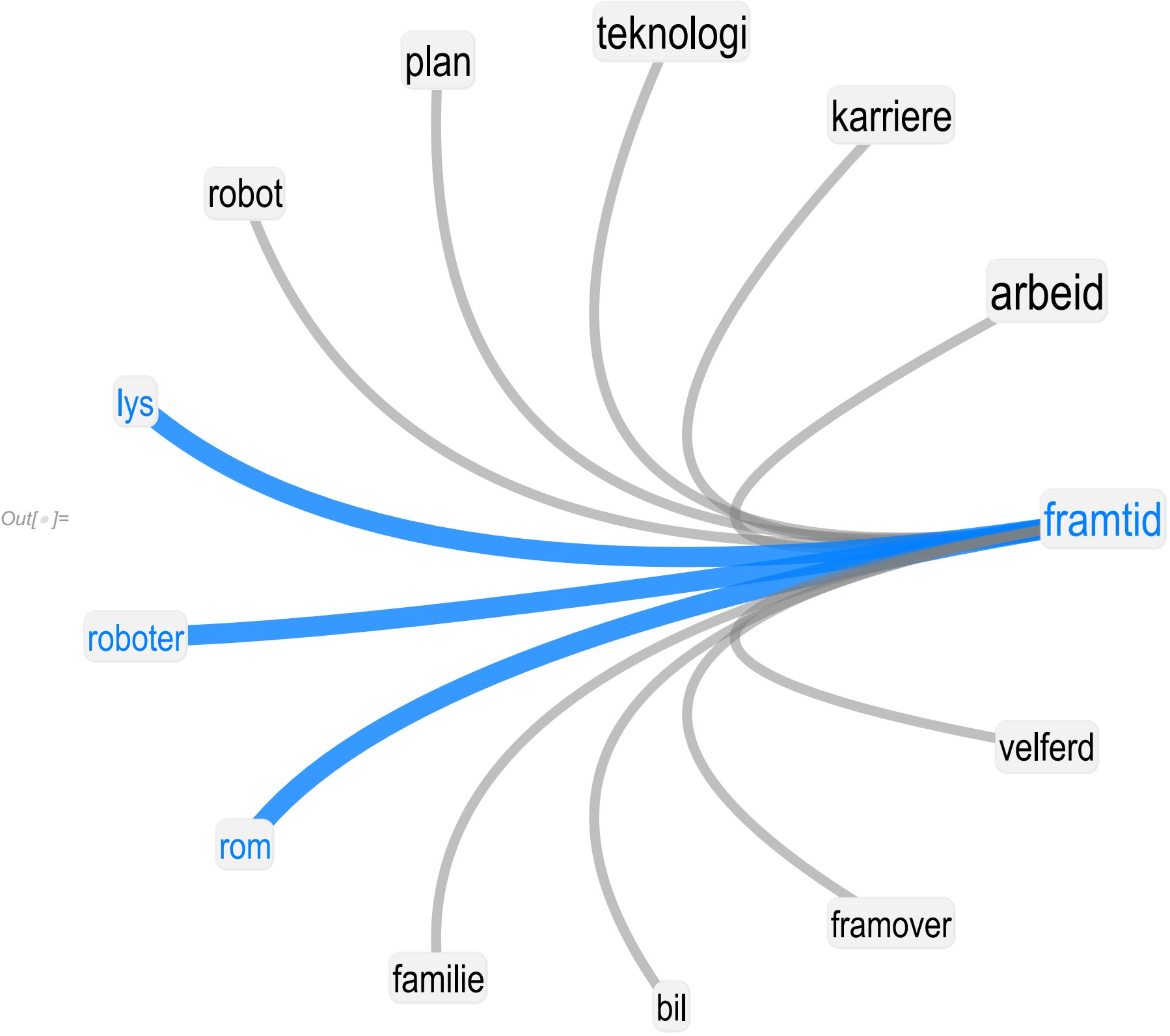

Supplement: Supplemental Information 1 — Every data file includes network links and valence attributes as reported in the main text. [file peerj-cs-06-255-s001.zip › Framtid Males.pdf]

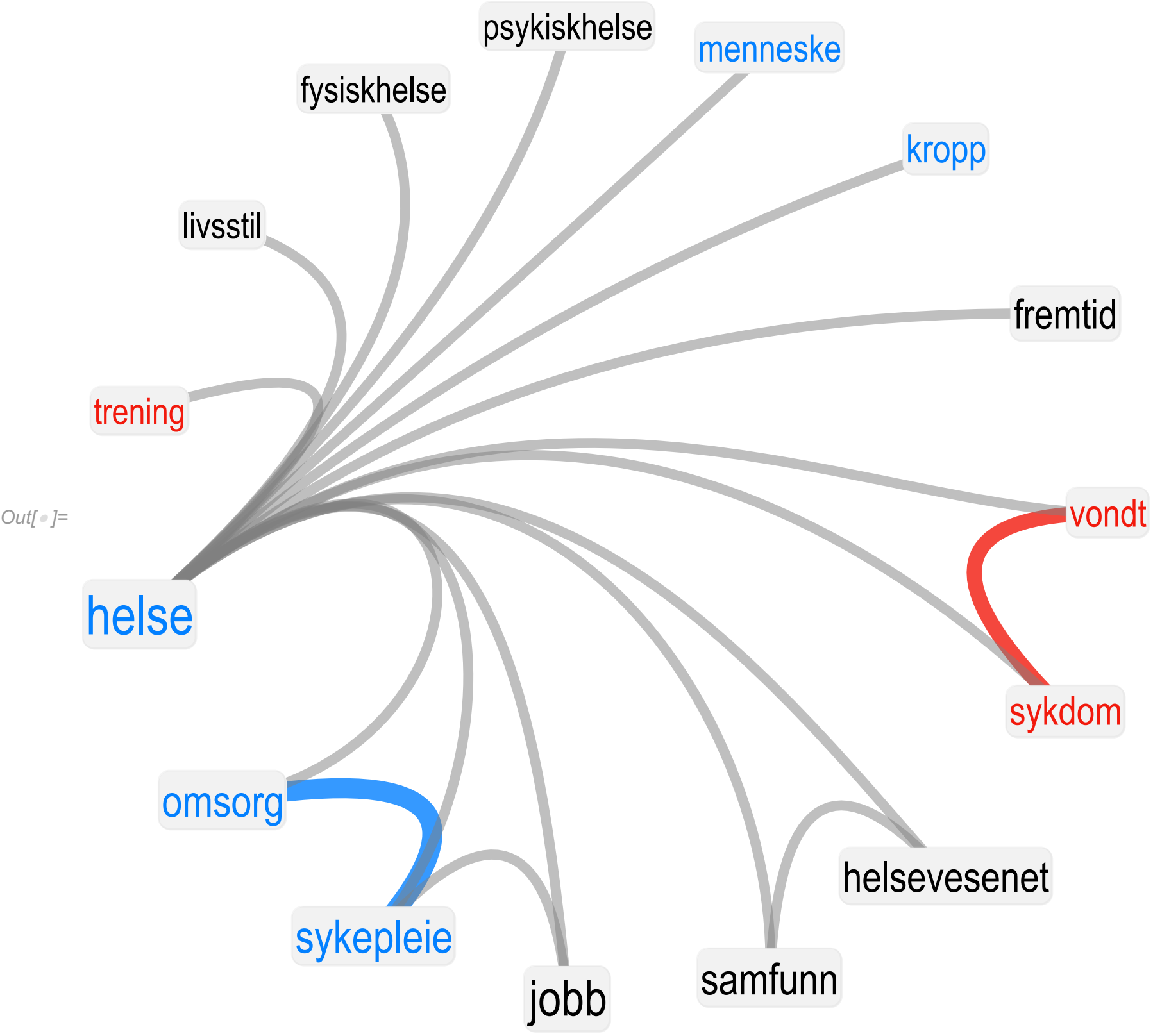

Supplement: Supplemental Information 1 — Every data file includes network links and valence attributes as reported in the main text. [file peerj-cs-06-255-s001.zip › Helse Females.pdf]

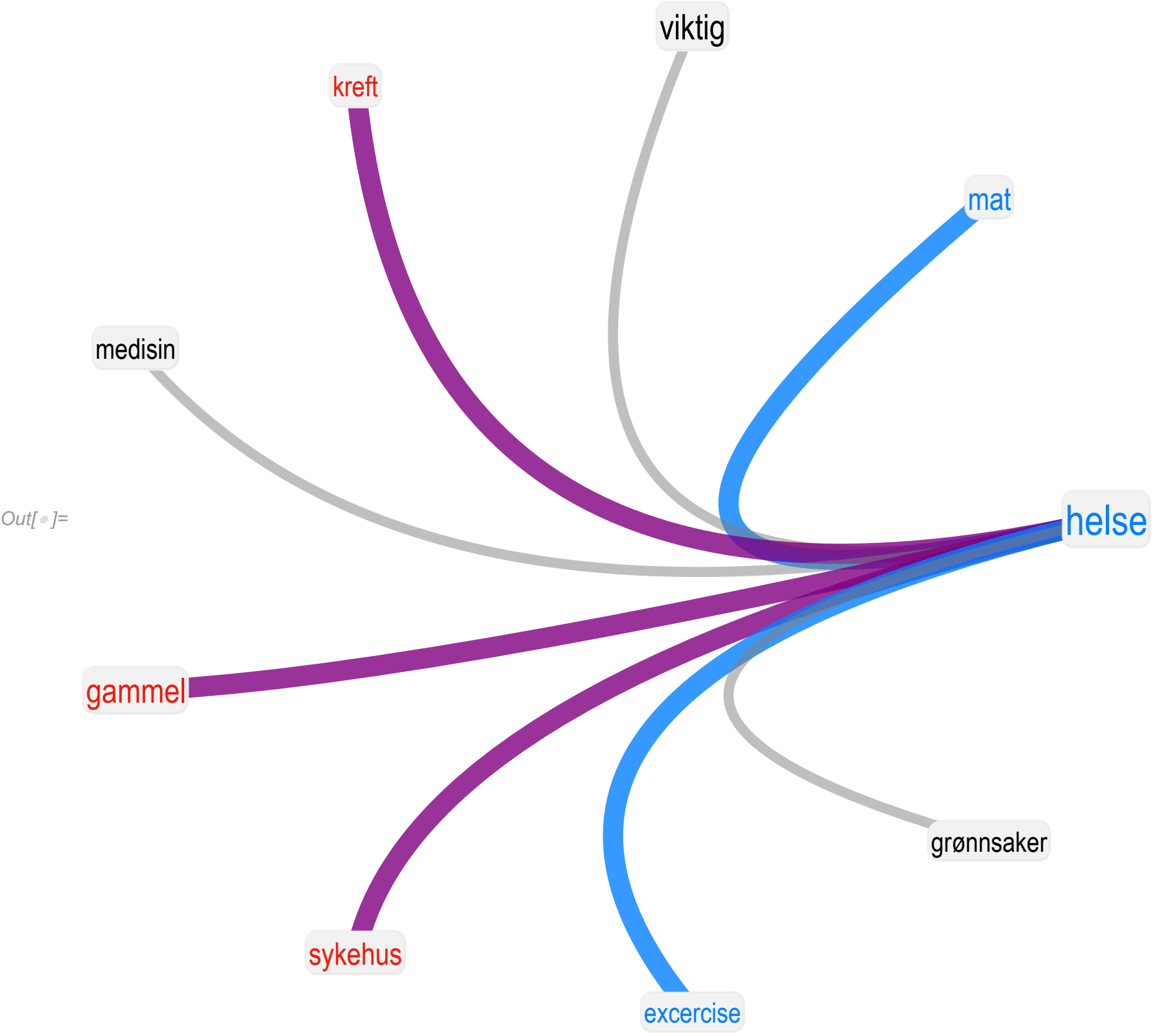

Supplement: Supplemental Information 1 — Every data file includes network links and valence attributes as reported in the main text. [file peerj-cs-06-255-s001.zip › Helse Males.pdf]

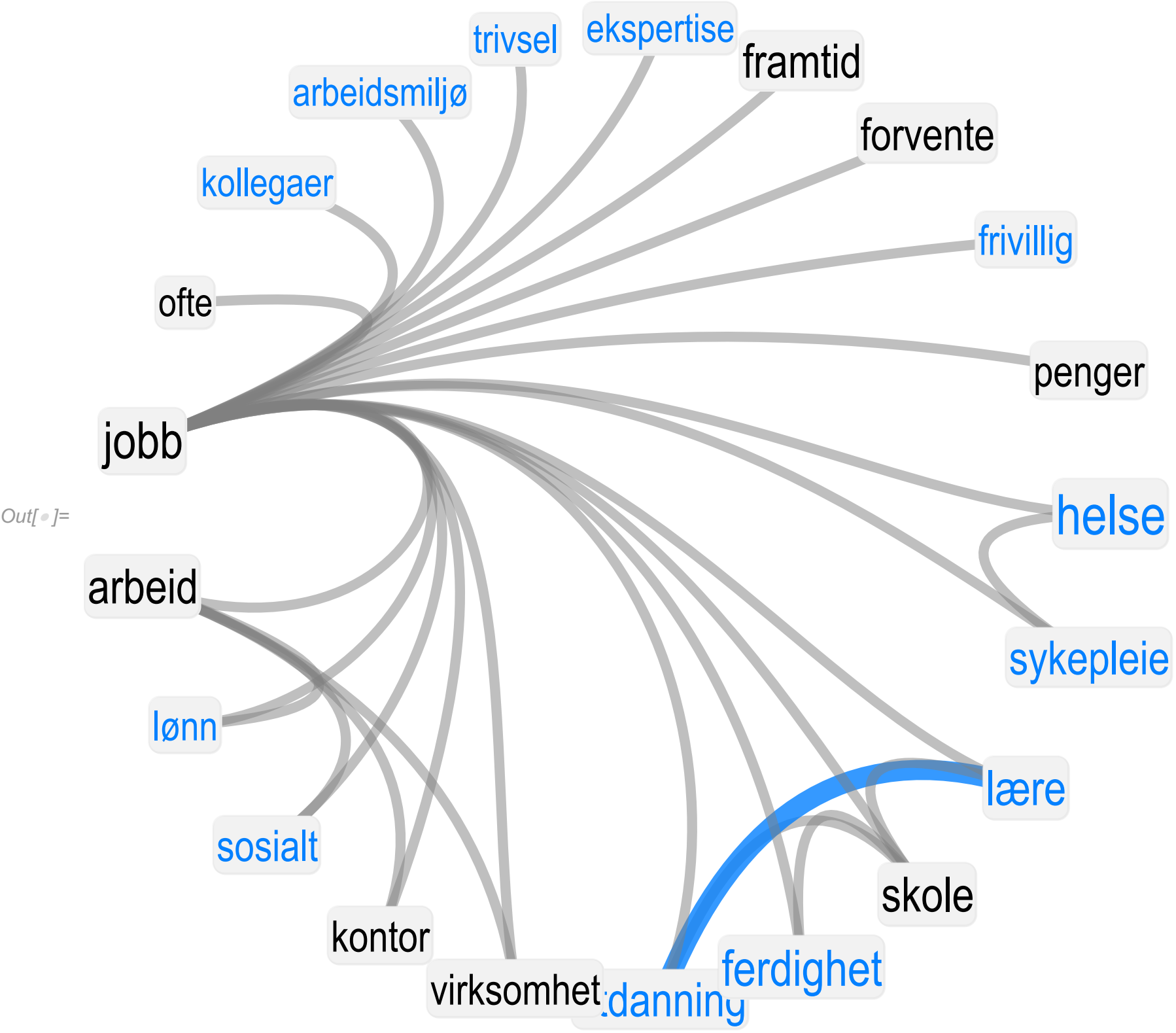

Supplement: Supplemental Information 1 — Every data file includes network links and valence attributes as reported in the main text. [file peerj-cs-06-255-s001.zip › Jobb Females.pdf]

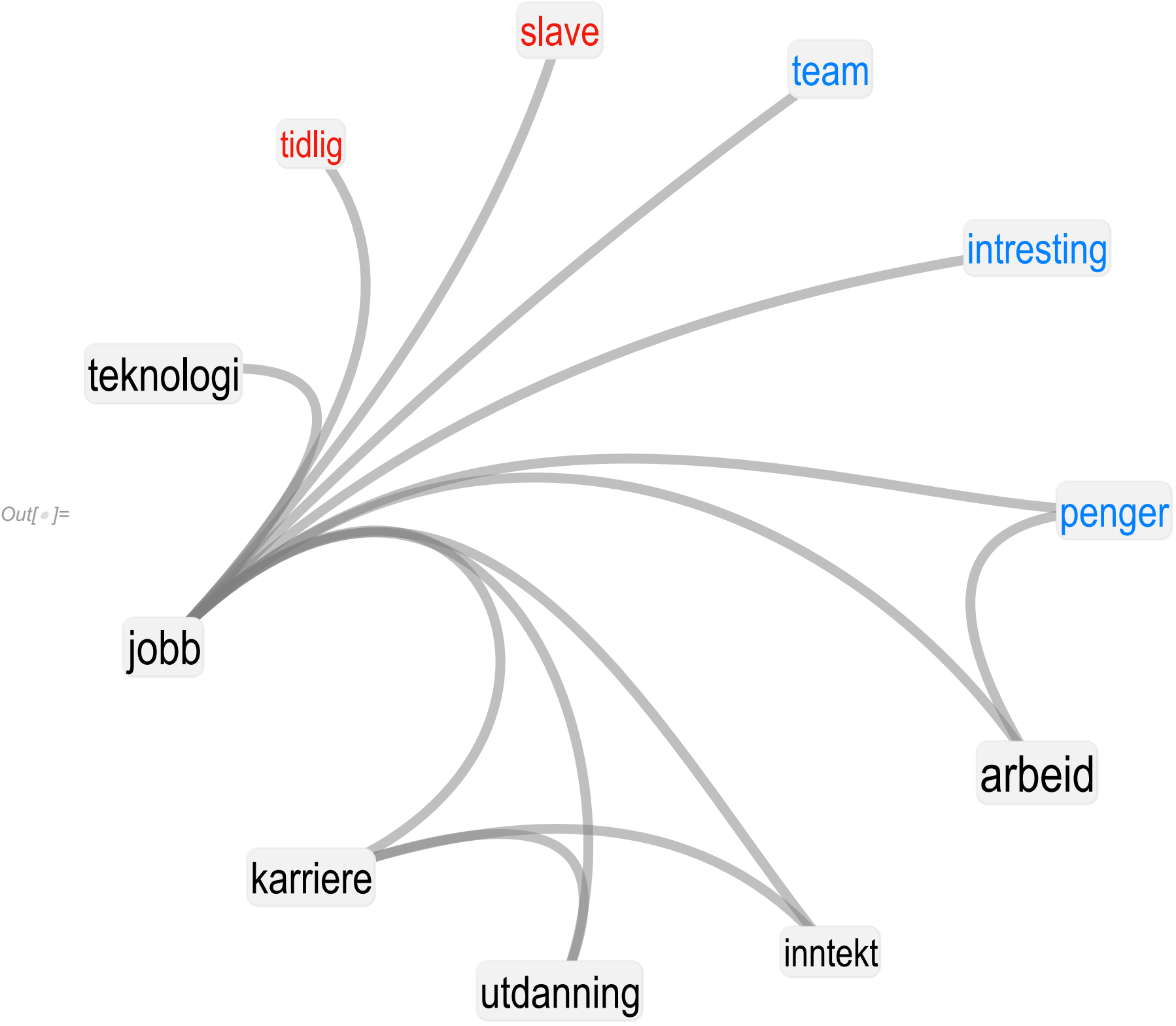

Supplement: Supplemental Information 1 — Every data file includes network links and valence attributes as reported in the main text. [file peerj-cs-06-255-s001.zip › Jobb Males.pdf]

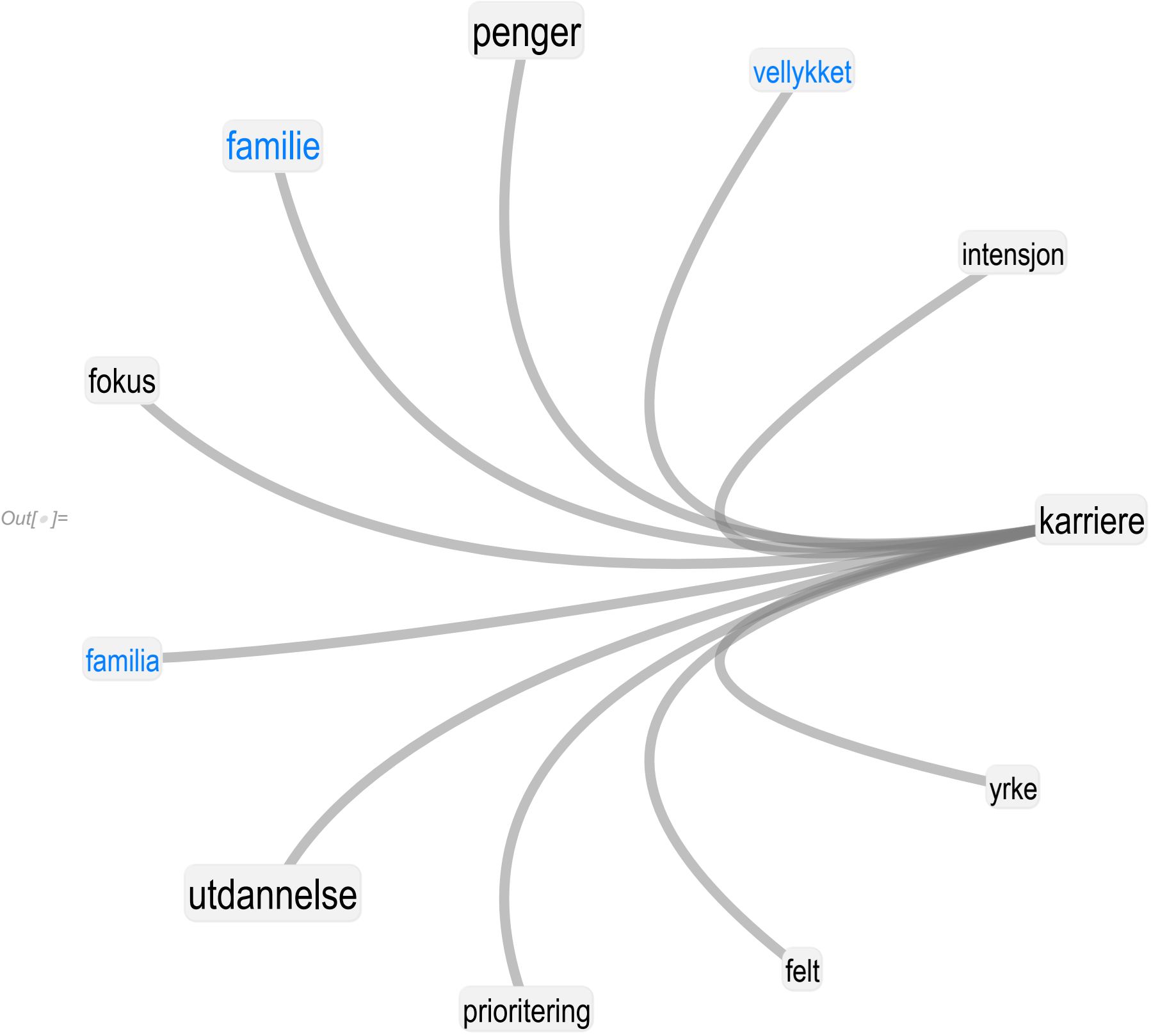

Supplement: Supplemental Information 1 — Every data file includes network links and valence attributes as reported in the main text. [file peerj-cs-06-255-s001.zip › Karriere Females.pdf]

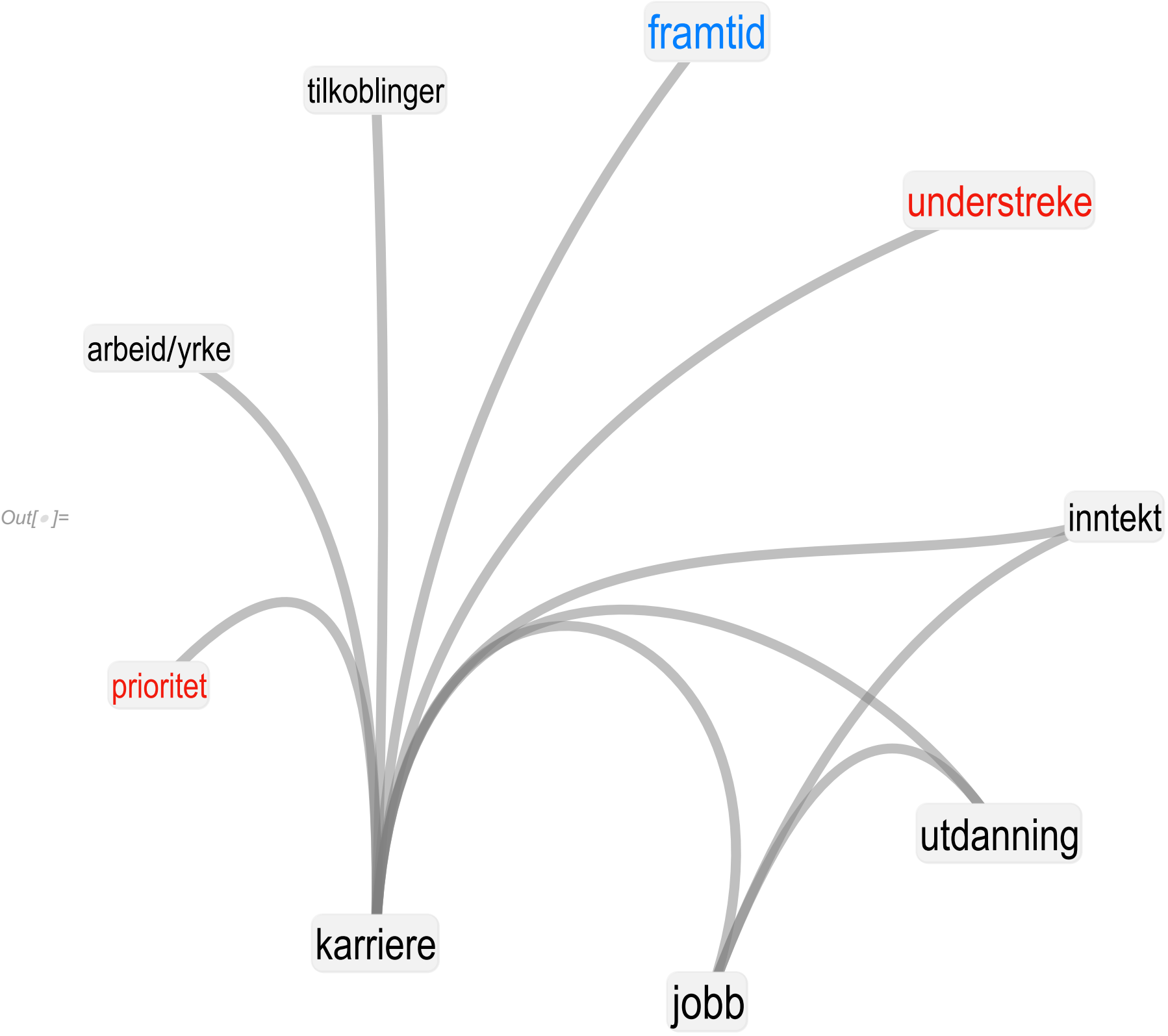

Supplement: Supplemental Information 1 — Every data file includes network links and valence attributes as reported in the main text. [file peerj-cs-06-255-s001.zip › Karriere Males.pdf]

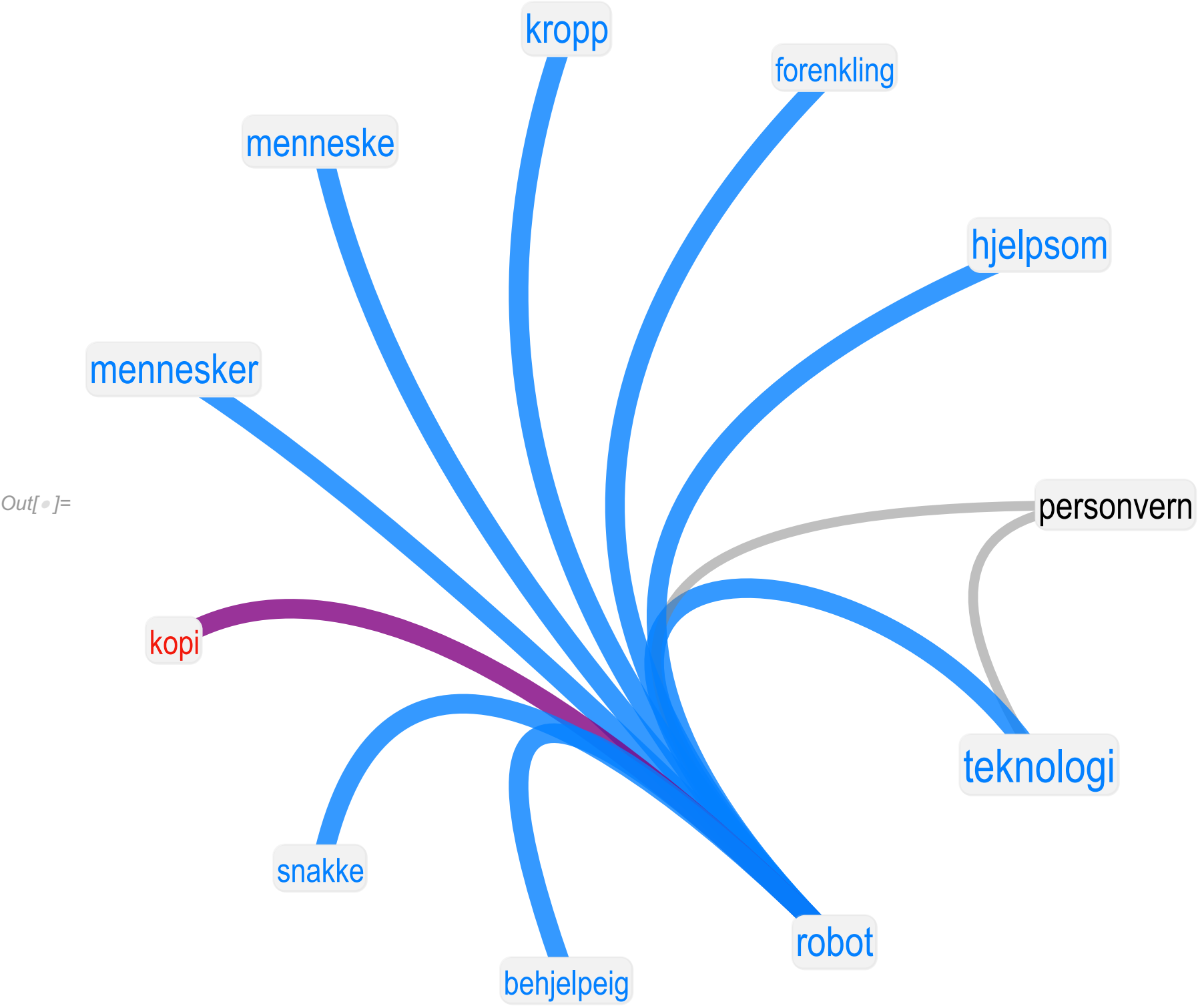

Supplement: Supplemental Information 1 — Every data file includes network links and valence attributes as reported in the main text. [file peerj-cs-06-255-s001.zip › Robot Females.pdf]

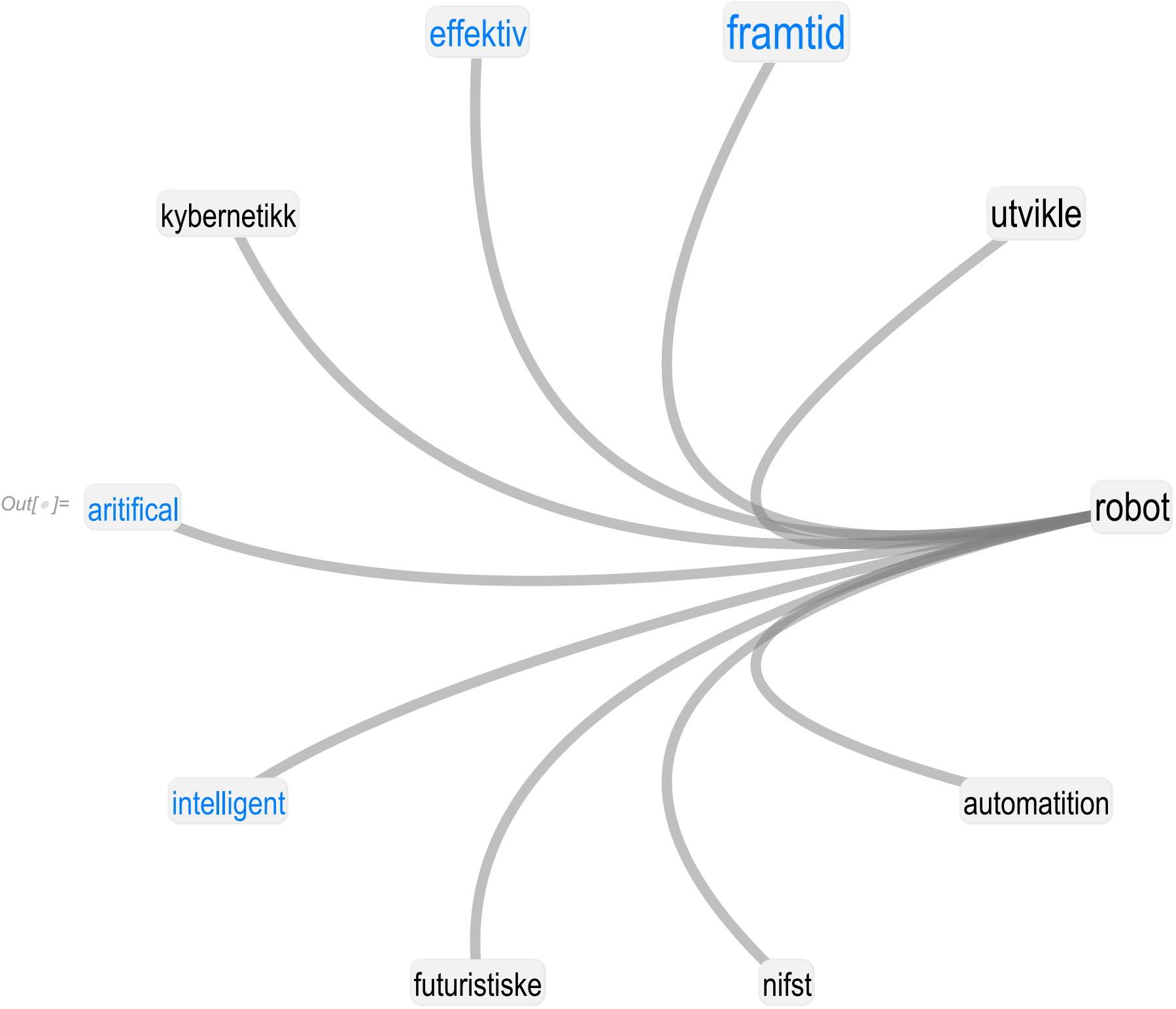

Supplement: Supplemental Information 1 — Every data file includes network links and valence attributes as reported in the main text. [file peerj-cs-06-255-s001.zip › Robot Males.pdf]

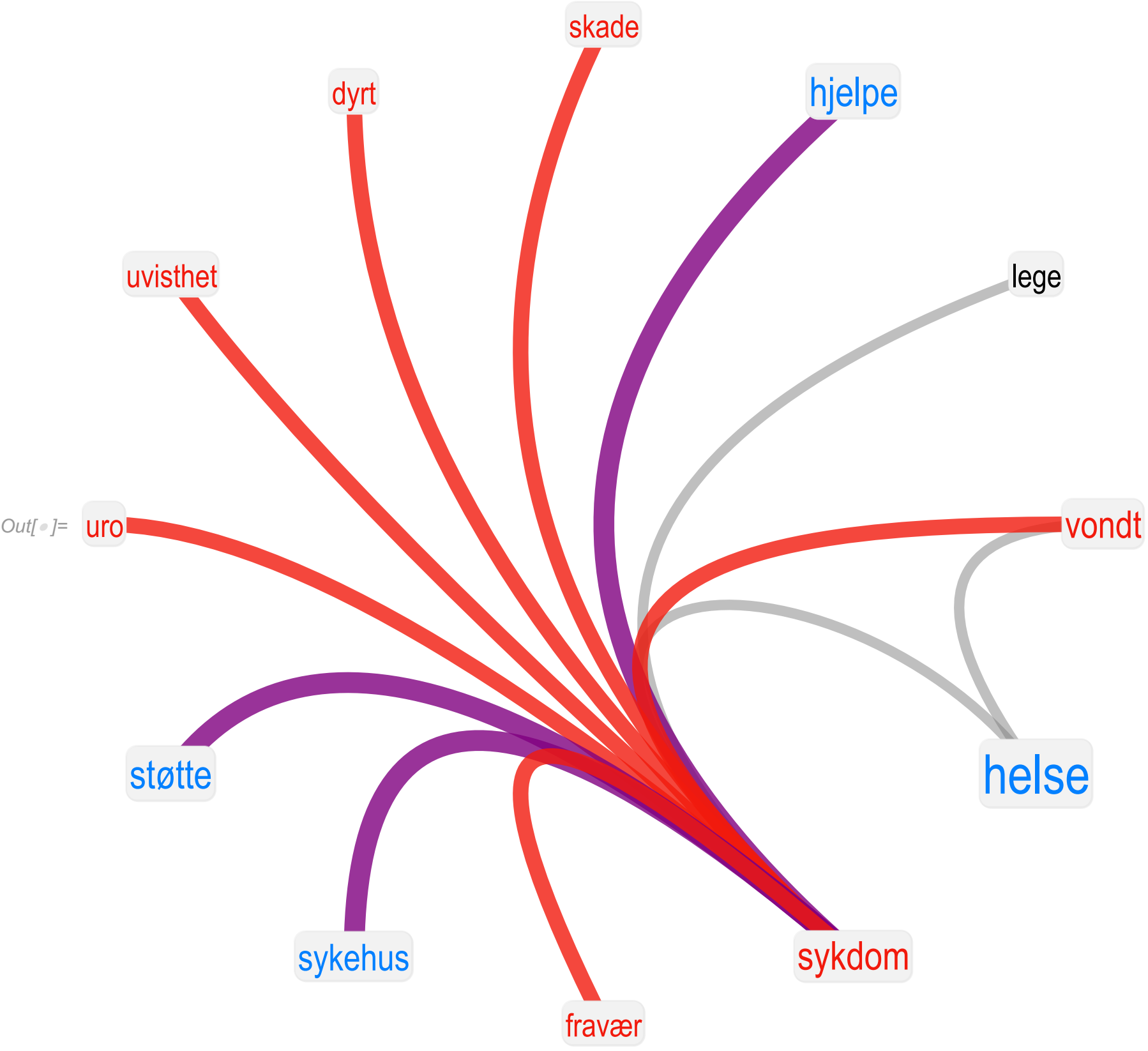

Supplement: Supplemental Information 1 — Every data file includes network links and valence attributes as reported in the main text. [file peerj-cs-06-255-s001.zip › Sykdom Females.pdf]

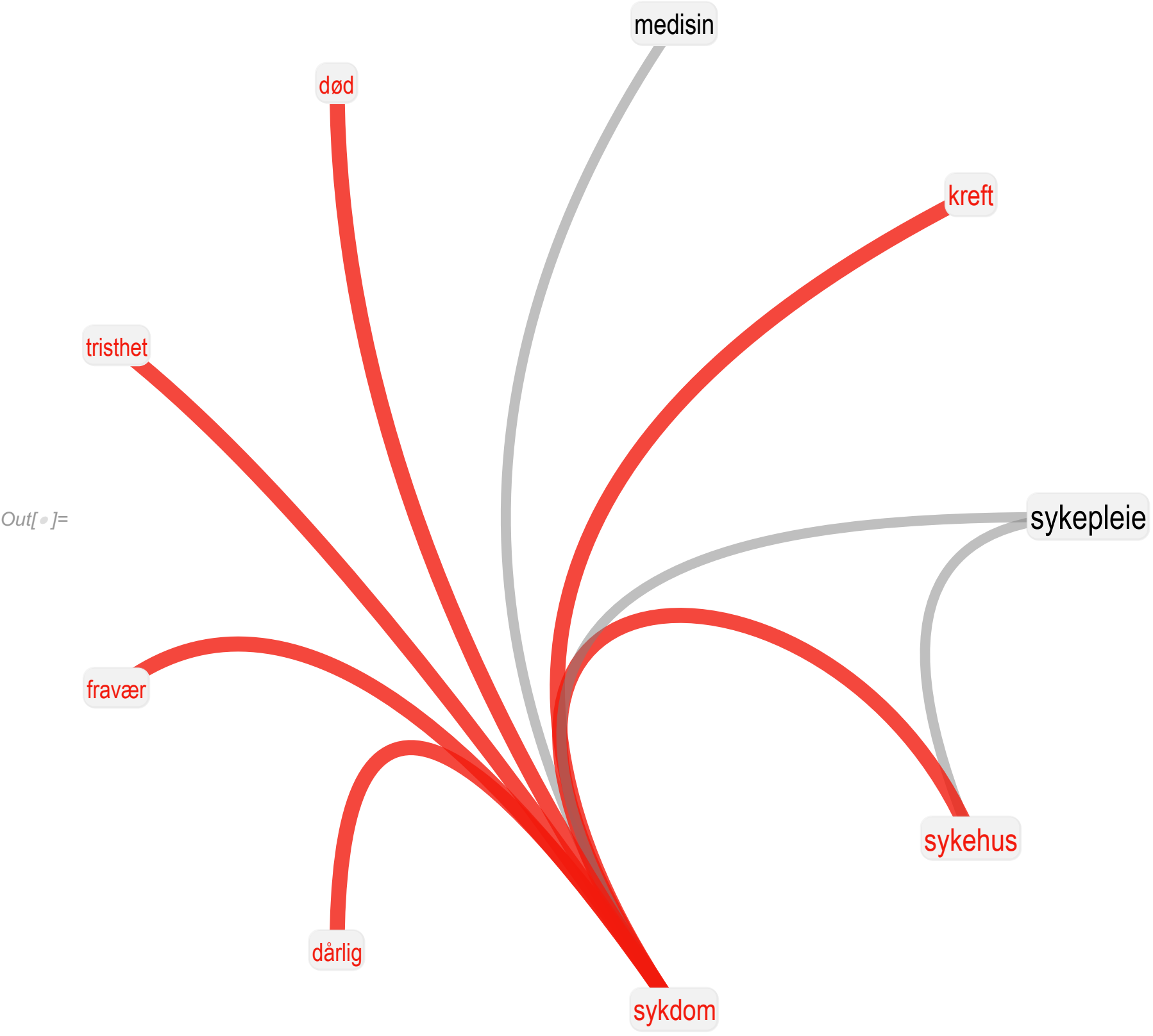

Supplement: Supplemental Information 1 — Every data file includes network links and valence attributes as reported in the main text. [file peerj-cs-06-255-s001.zip › Sykdom Males.pdf]

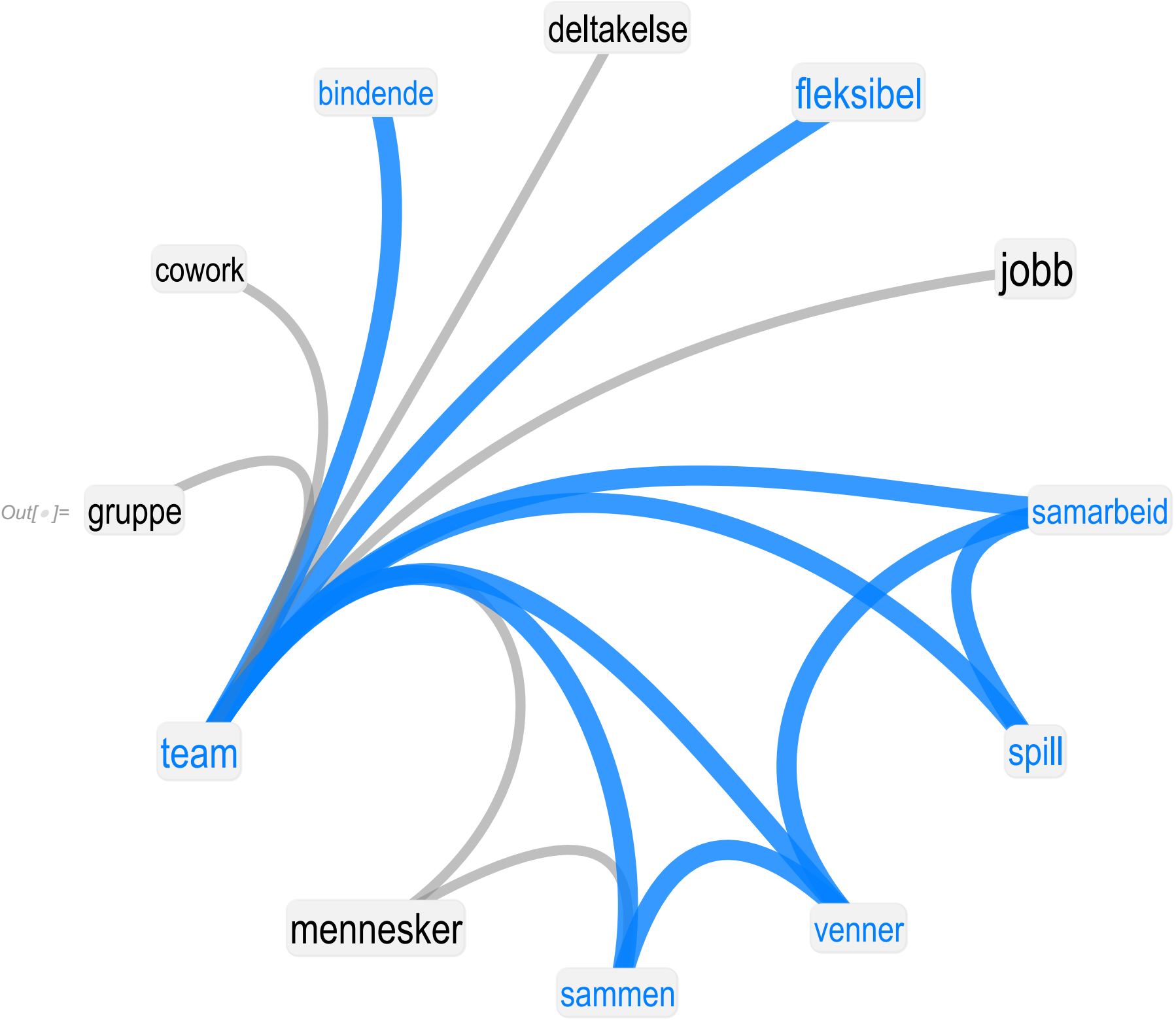

Supplement: Supplemental Information 1 — Every data file includes network links and valence attributes as reported in the main text. [file peerj-cs-06-255-s001.zip › Team Males.pdf]

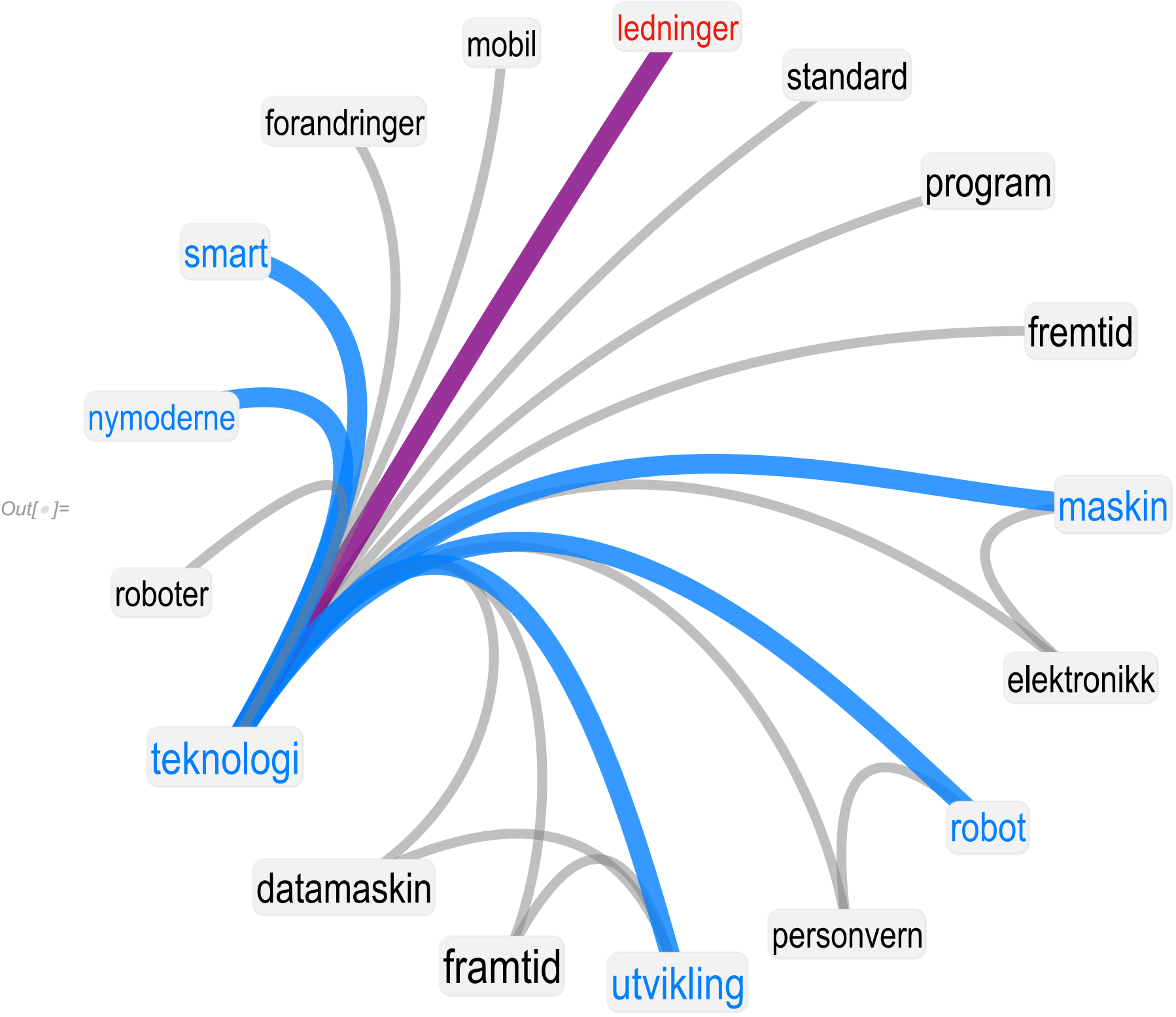

Supplement: Supplemental Information 1 — Every data file includes network links and valence attributes as reported in the main text. [file peerj-cs-06-255-s001.zip › Teknologi Females.pdf]

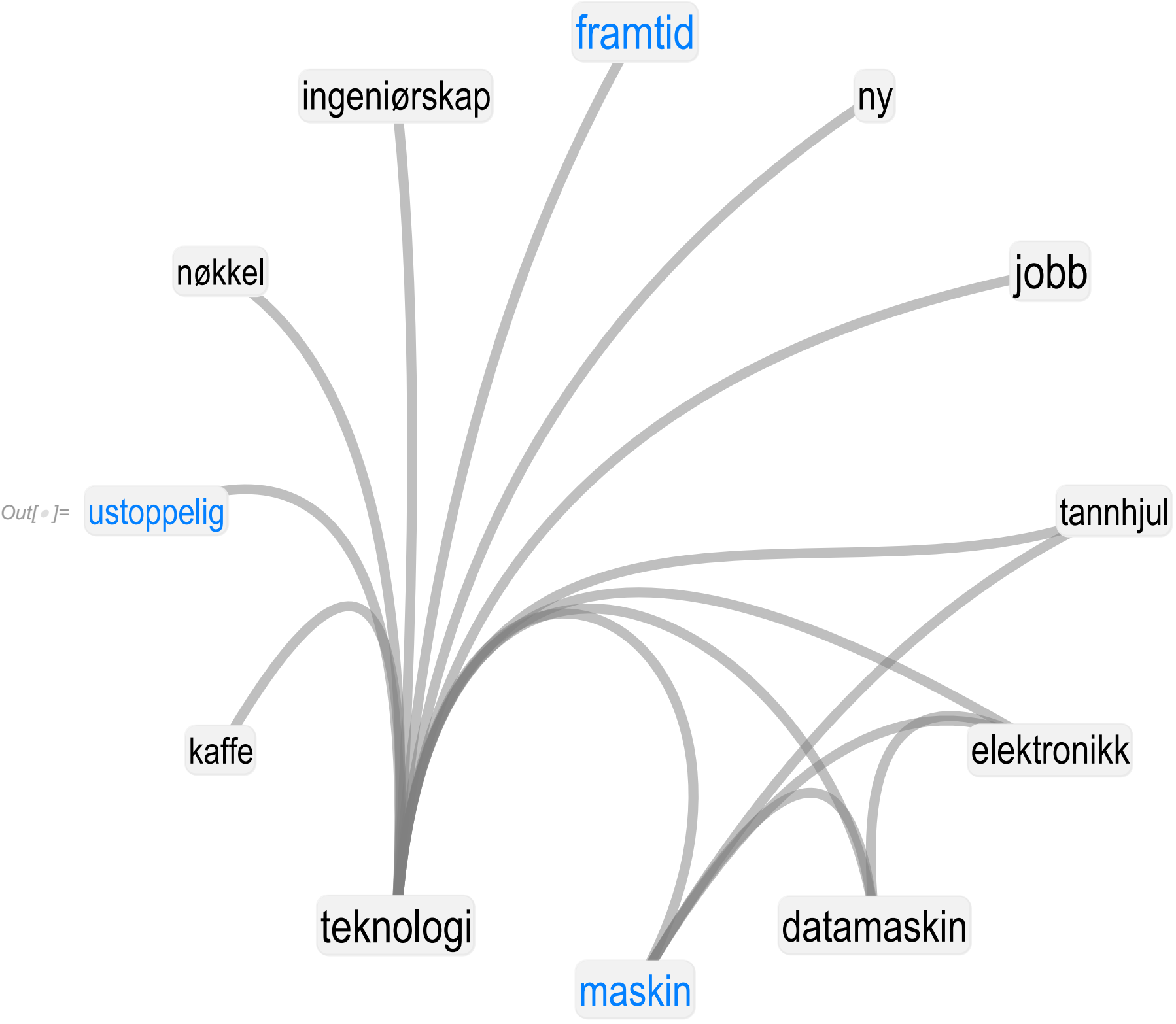

Supplement: Supplemental Information 1 — Every data file includes network links and valence attributes as reported in the main text. [file peerj-cs-06-255-s001.zip › Teknologi Males.pdf]
