# Supplementary material for: Forma mentis networks map how nursing and engineering students enhance their mindsets about innovation and health during professional growth
Source: PeerJ Comput Sci. 2020 Mar 2;6:e255. doi: 10.7717/peerj-cs.255 (PMC7924483; doi:10.7717/peerj-cs.255)
Supplement: Supplemental Information 2 [file peerj-cs-06-255-s002.pdf]

Supplementary Information

Forma mentis networks map how nursing and engineering students enhance their mindsets about innovation and health during professional growth

Massimo Stella and Anna Zaytseva

The following Appendix contains additional figures about local changes in the forma mentis of students before and after the summer job experience.

Figure S1 - Forma mentis networks for Student 1 (nursing student) before (top) and after (bottom) the summer job experience. Negative/positive/neutral words are highlighted in red, cyan and black, respectively. Links between positive (negative) words are highlighted in cyan (red). Purple links indicate associations between positive and negative concepts.

Figure S2 - Forma mentis networks for Student 2 (nursing student) before (top) and after (bottom) the summer job experience. Negative/positive/neutral words are highlighted in red, cyan and black, respectively. Links between positive (negative) words are highlighted in cyan (red). Purple links indicate associations between positive and negative concepts.

Figure S3 - Forma mentis networks for Student 3 (nursing student) before (top) and after (bottom) the summer job experience. Negative/positive/neutral words are highlighted in red, cyan and black, respectively. Links between positive (negative) words are highlighted in cyan (red). Purple links indicate associations between positive and negative concepts.

Figure S4 - Forma mentis networks for Student 4 (engineering student) before (top) and after (bottom) the summer job experience. Negative/positive/neutral words are highlighted in red, cyan and black, respectively. Links between positive (negative) words are highlighted in cyan (red). Purple links indicate associations between positive and negative concepts.

Figure S5 - Forma mentis networks for Student 5 (engineering student) before (top) and after (bottom) the summer job experience. Negative/positive/neutral words are highlighted in red, cyan and black, respectively. Links between positive (negative) words are highlighted in cyan (red). Purple links indicate associations between positive and negative concepts.

Figure S6 - Forma mentis networks for Student 6 (engineering student) before (top) and after (bottom) the summer job experience. Negative/positive/neutral words are highlighted in red, cyan and black, respectively. Links between positive (negative) words are highlighted in cyan (red). Purple links indicate associations between positive and negative concepts.

Figure S7 - Cumulative degree distribution for the “before”, “after” and “core” forma mentis networks, as described in the main paper. The distribution P(X) indicates the probability of finding a node with degree k equal to or greater than X in a given network. The degree distributions of the “before” and “after” networks are quite similar and exhibit a heavy tail.

Supplementary Text S1 - Description of the Intervention Event
Supplementary Text S2 - Qualitative Feedback from Students
Supplementary Table S1 - List of Cue Words

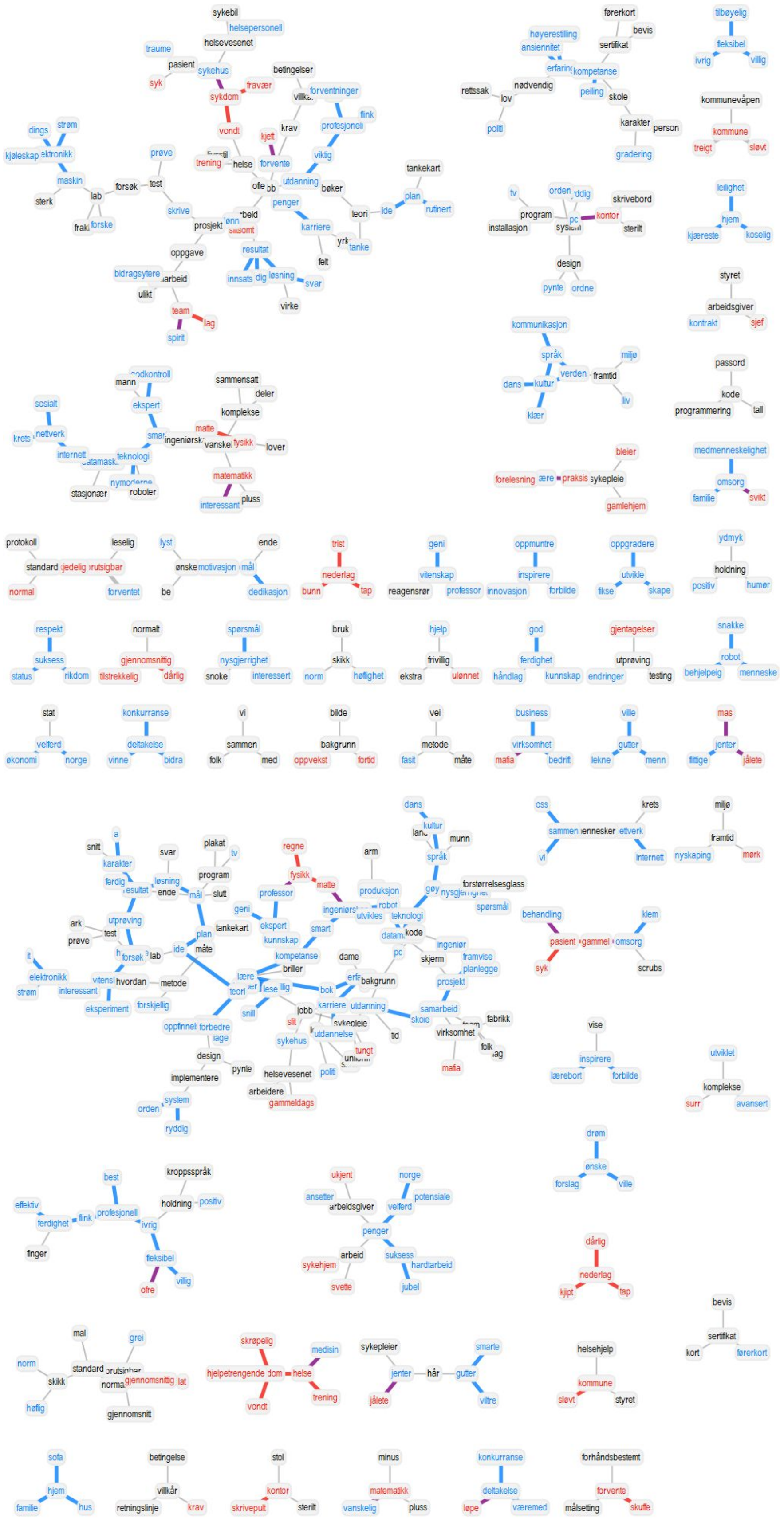
tilbøyelig
fleksibel
ivrig
villig
kommunevåpen
kommune
treigt
sløvt
leilighet
hjem
kjæreste
koselig
styret
arbeidsgiver
kontrakt
sjef
passord
kode
programmering
tall
medmenneskelighet
omsorg
familie
svikt
ydmyk
holdning
positiv
humør
snakke
robot
behjelpelig
menneske
trist
nederlag
bunn
tap
geni
vitenskap
reagensrør
professor
oppmuntre
inspirere
innovasjon
forbilde
oppgradere
utvikle
fikse
skape
respekt
suksess
status
rikdom
normalt
gjennomsnittig
tilstrekkelig
dårlig
spørsmål
nysgjerrighet
snoke
interessert
bruk
skikk
norm
høflighet
hjelp
frivillig
ekstra
ulønnet
god
ferdighet
håndlag
kunnskap
gjentagelser
utprøving
endringer
testing
stat
velferd
økonomi
norge
konkurranse
deltakelse
vinne
bidra
vi
sammen
folk
med
bilde
bakgrunn
oppvekst
fortid
vei
metode
fasit
måte
business
virksomhet
mafia
bedrift
ville
gutter
lekne
menn
mas
jenter
flittige
jålete
vise
inspirere
lærebort
forbilde
utviklet
komplekse
surr
avansert
drøm
ønske
forslag
ville
dårlig
nederlag
kjipt
tap
bevis
sertifikat
kort
førerkort
helsehjelp
kommune
sløvt
styret
sykepleier
jenter
hår
gutter
smarte
jålete
viltre
sofa
hjem
familie
hus
betingelse
villkår
retningslinje
krav
stol
kontor
skrivepult
sterilt
minus
matematikk
vanskelig
pluss
konkurranse
deltakelse
løpe
væremed
forhåndsbestemt
forvente
målsetting
skuffe

Figure S1

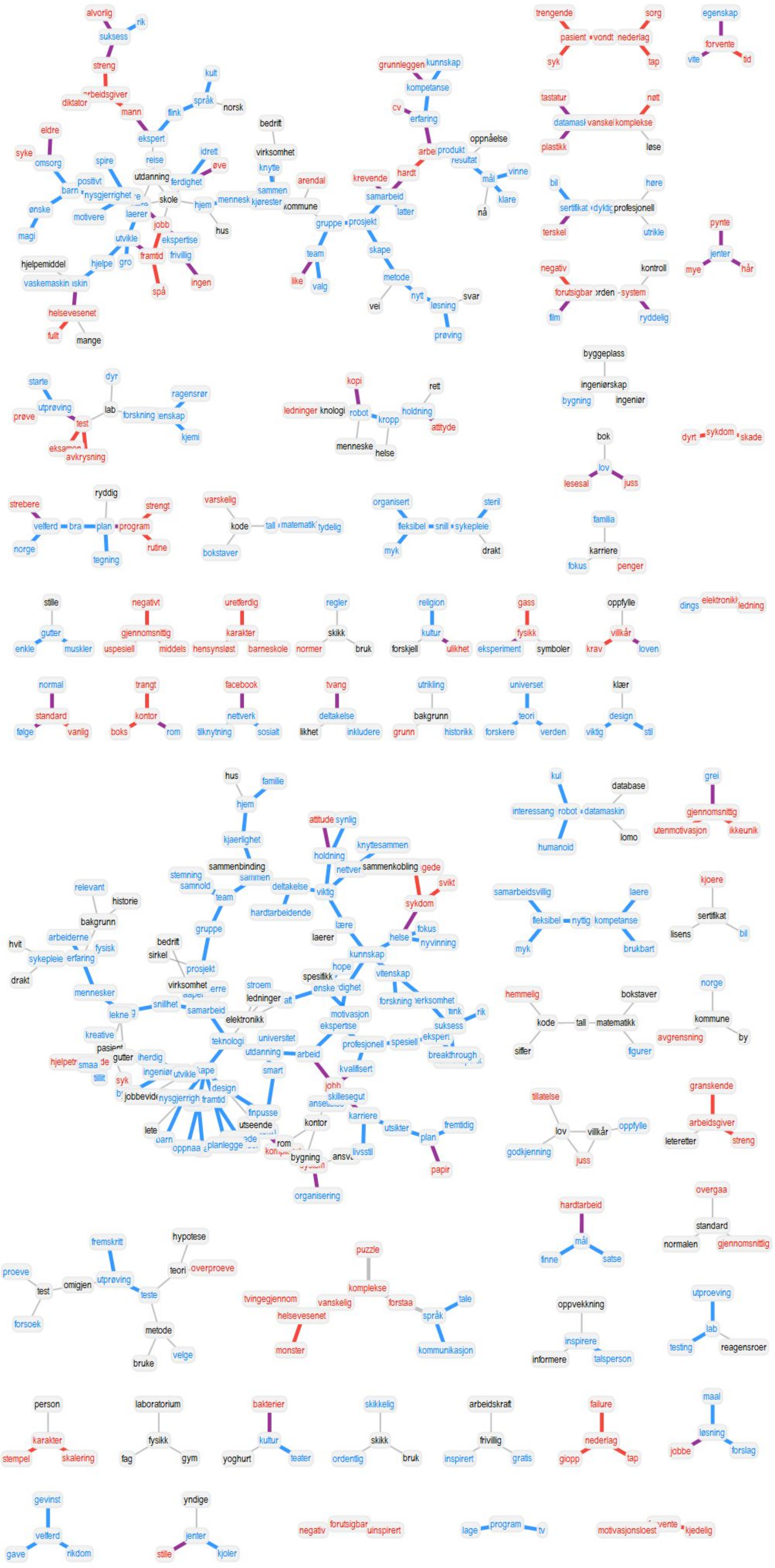

alvorlig
rik
suksess
streng
arbeidsgiver
diktator
mann
ekspert
kult
språk
norsk
flink
eldre
syke
omsorg
spire
reise
idrett
øve
utdanning
ferdighet
skole
hjem
hus
positivt
barn
nysgjerrighet
ønske
motivere
laerer
jobb
magi
utvikle
ekspertise
hjelpemiddel
hjelpe
gro
framtid
frivillig
vaskemaskin
spå
ingen
helsevesenet
fullt
mange
bedrift
virksomhet
knytte
sammen
arendal
kommune
gruppe
prosjekt
team
like
valg
skape
metode
vei
nytt
løsning
svar
prøving
grunnleggende
kunnskap
kompetanse
cv
erfaring
oppnåelse
produkt
resultat
hardt
krevende
samarbeid
later
mål
vinne
klare
nå
trengende
sorg
pasient
vondt
nederlag
syk
tap
egenskap
forvente
vite
tid
tastatur
nøtt
vanskelig
komplekse
plastikk
løse
bil
høre
sertifikat
profesjonell
terskel
utrikle
pynte
jenter
mye
hår
negativ
kontroll
forutsigbar
system
film
ryddelig
byggeplass
ingeniørskap
bygning
ingeniør
bok
lov
lesesal
juss
dyrt
sykdom
skade
familia
karriere
fokus
penger
starte
dyr
utprøving
lab
reagensrør
prøve
test
forskning
kjemi
avkrysning
kopi
rett
ledninger
robot
holdning
kropp
attityde
menneske
helse
ryddig
strebere
strengt
velferd
bra
plan
program
norge
rutine
tegning
varskelig
kode
tall
matematikk
tydelig
bokstaver
organisert
steril
fleksibel
snill
sykepleie
myk
drakt
stille
gutter
enkle
muskler
negativt
gjennomsnittig
uspesiell
middels
urettferdig
karakter
hensynsløst
barneskole
regler
skikk
normer
bruk
religion
kultur
forskjell
ulikhet
gass
fysikk
eksperiment
symboler
oppfylle
villkår
krav
loven
dings
elektronikk
ledning
normal
standard
følge
vanlig
trangt
kontor
boks
rom
facebook
nettverk
tilknytning
sosialt
tvang
deltakelse
likhet
inkludere
utrikling
bakgrunn
grunn
historikk
universet
teori
forskere
verden
klær
design
viktig
stil
familie
kjaerlighet
sammenbinding
attitude
synlig
knyttesammen
sammenkobling
gede
svikt
hardtarbeidende
laerer
lære
fokus
nyvinning
kunnskap
relevant
historie
hvit
drakt
bedrift
sirkel
kul
database
interessang
datamaskin
humanoid
lomo
grei
utenmotivasjon
ikkeunik
kjoere
samarbeidsvillig
laere
nyttig
brukbart
lisens
hemmelig
bokstaver
siffer
matematikk
figurer
kommune
avgrensning
by
tillatelse
villkår
godkjenning
granskende
leteretter
hardtarbeid
finne
satse
overgaa
normalen
fremskritt
hypotese
proeve
omigjen
teori
overproeve
teste
forsoek
bruke
velge
puzzle
tvingegjennom
forstaa
tale
monster
kommunikasjon
oppvekkning
inspirere
informere
talsperson
utproeving
testing
reagensroer
person
stempel
skalering
laboratorium
fag
gym
bakterier
yoghurt
teater
skikkelig
ordentlig
arbeidskraft
frivillig
inspirert
gratis
failure
giopp
maal
jobbe
forslag
gevinst
gave
rikdom
yndige
kjoler
uinspirert
lage
tv
motivasjonsloest
kjedelig

Figure S2

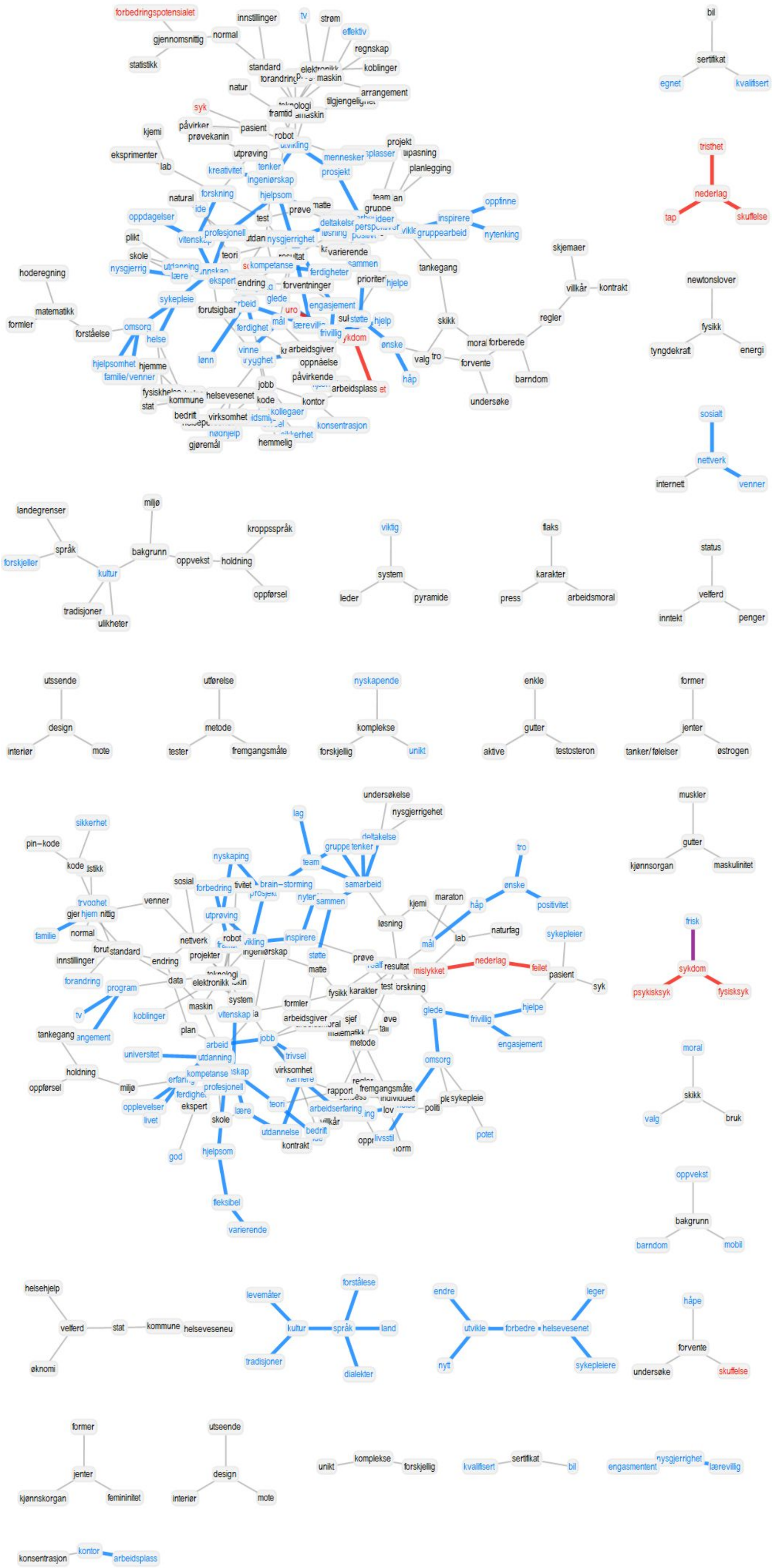

forbedringspotensialet
gjennomsnittig
statistikk
normal
innstillinger
tv
strøm
effektiv
regnskap
standard
koblinger
maskin
arrangement
natur
tilgjengelighet
syk
påvirker
prøvekanin
pasient
robot
kjemi
eksprimenter
lab
utprøving
kreativitet
tenker
ingeniørskap
mennesker
prosjekt
projekt
tilpasning
planlegging
natural
forskning
hjelpsom
test
prøve
matte
gruppe
team
oppdagelser
profesjonell
nysgjerrighet
inspirere
oppfinne
gruppearbeid
nytenking
plikt
skole
teori
variererende
nysgjerrig
utdanning
lære
ekspert
kompetanse
endring
forventninger
ferdigheter
sammen
prioriter
hjelpe
tankegang
skjemaer
hoderegning
matematikk
formler
forståelse
sykepleie
forutsigbar
engasjement
uro
glede
ferdighet
lærevillig
frivillig
støtte
hjelp
skikk
regler
villkår
kontrakt
omsorg
helse
sykdom
ønske
moral
forberede
hjelpsomhet
hjemme
familie/venner
lønn
vinne
arbeidsgiver
oppnåelse
valg
tro
forvente
håp
barndom
påvirkende
jobb
arbeidsplass
fysiskhelse
kommune
helsevesenet
kode
kontor
undersøke
stat
bedrift
virksomhet
kollegaer
konsentrasjon
gjøremål
hemmelig
bil
sertifikat
egnet
kvalifisert
tristhet
nederlag
tap
skuffelse
newtonslover
fysikk
tyngdekraft
energi
sosialt
nettverk
internett
venner
landegrenser
miljø
språk
bakgrunn
oppvekst
holdning
kroppsspråk
forskjeller
kultur
tradisjoner
ulikheter
oppførsel
viktig
system
leder
pyramide
flaks
karakter
press
arbeidsmoral
status
velferd
inntekt
penger
utssende
design
interiør
mote
utførelse
metode
tester
fremgangsmåte
nyskapende
komplekse
forskjellig
unikt
enkle
gutter
aktive
testosteron
former
jenter
tanker/følelser
østrogen
undersøkelse
nysgjerrighet
lag
sikkerhet
pin−kode
nyskaping
deltakelse
samarbeid
brain−storming
tro
ønske
positivitet
sosial
forbedring
venner
familie
innstillinger
forandring
program
tv
koblinger
nettverk
robot
projekter
endring
elektronikk
data
system
maskin
plan
inspirere
løsning
kjemi
maraton
naturfag
sykepleier
lab
mål
nederlag
mislykket
feilet
pasient
syk
resultat
formler
arbeidsgiver
fysikk
sjef
øve
matematikk
glede
frivillig
hjelpe
engasjement
tankegang
holdning
oppførsel
miljø
universitet
utdanning
arbeid
jobb
trivsel
omsorg
kompetanse
virksomhet
karriere
rapport
fremgangsmåte
individuelt
sykepleie
opplevelser
livet
ekspert
erfaring
ferdighet
profesjonell
teori
arbeidserfaring
lov
politi
potet
skole
lære
utdannelse
bedrift
villkår
kontrakt
livsstil
norm
god
hjelpsom
fleksibel
varierende
muskler
gutter
kjønnsorgan
maskulinitet
frisk
sykdom
psykisksyk
fysisksyk
moral
skikk
valg
bruk
oppvekst
bakgrunn
barndom
mobil
helsehjelp
velferd
stat
kommune
helseveseneu
øknomi
levemåter
forståelse
kultur
språk
land
tradisjoner
dialekter
endre
utvikle
forbedre
helsevesenet
leger
nytt
sykepleiere
håpe
forvente
undersøke
skuffelse
former
jenter
kjønnskorgan
femininitet
utseende
design
interiør
mote
unikt
komplekse
forskjellig
kvalifisert
sertifikat
bil
engasmentent
nysgjerrighet
lærevillig
konsentrasjon
kontor
arbeidsplass

Figure S3

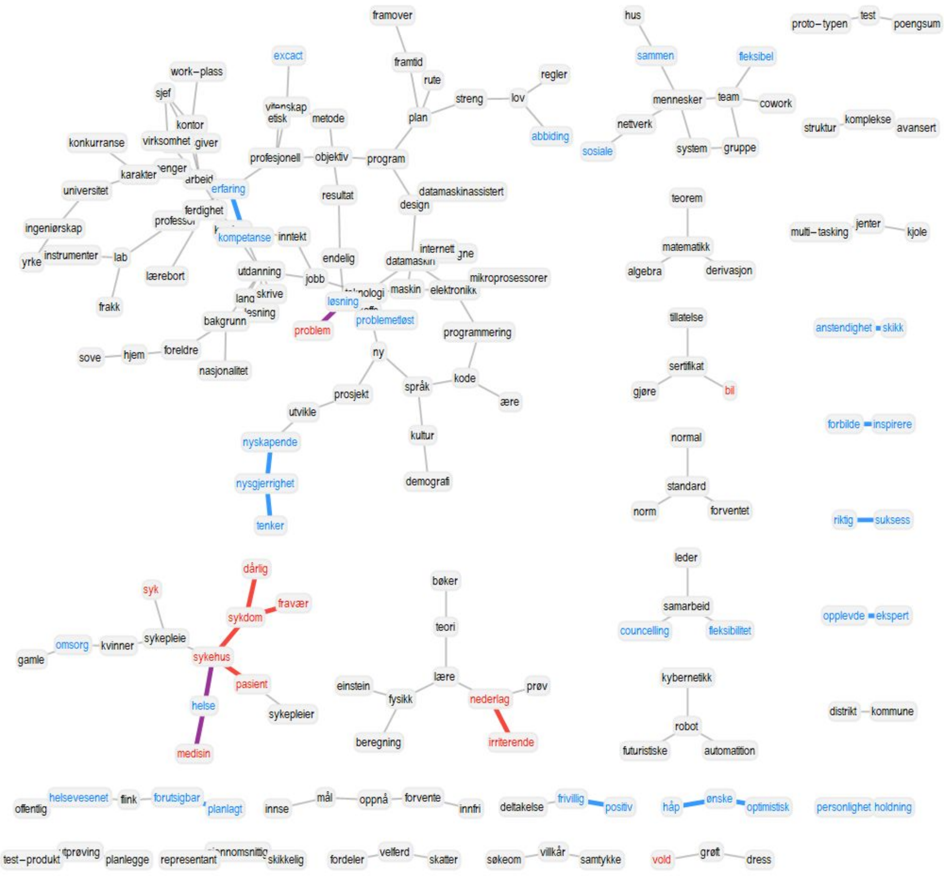

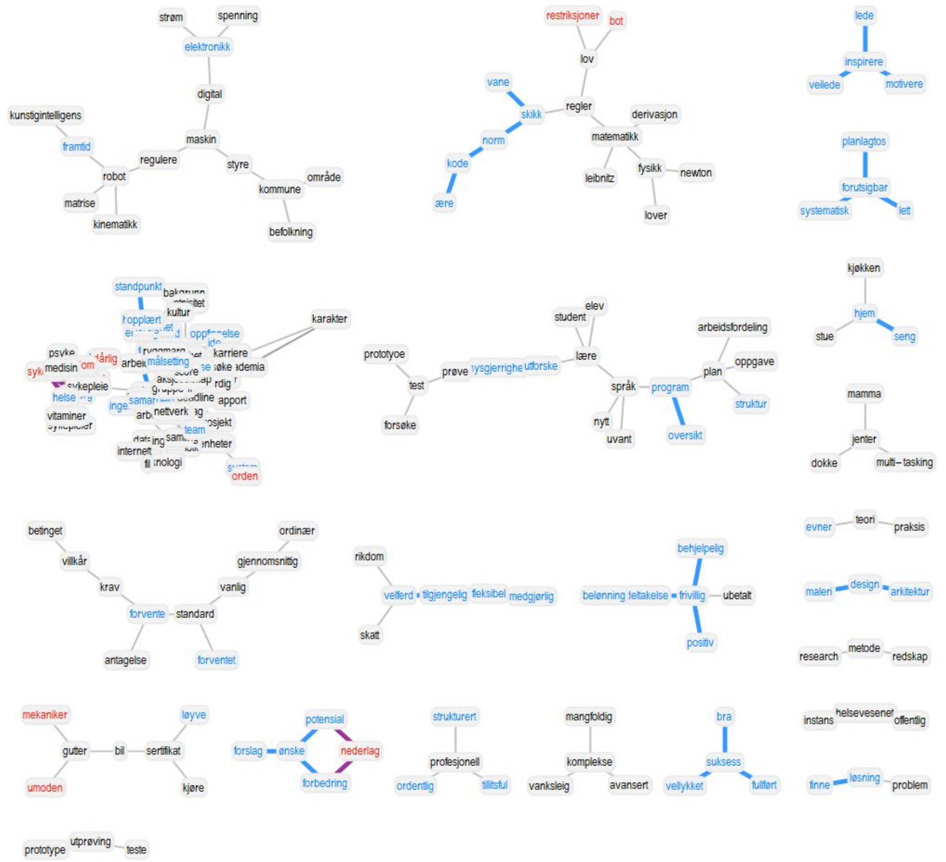

Figure S4

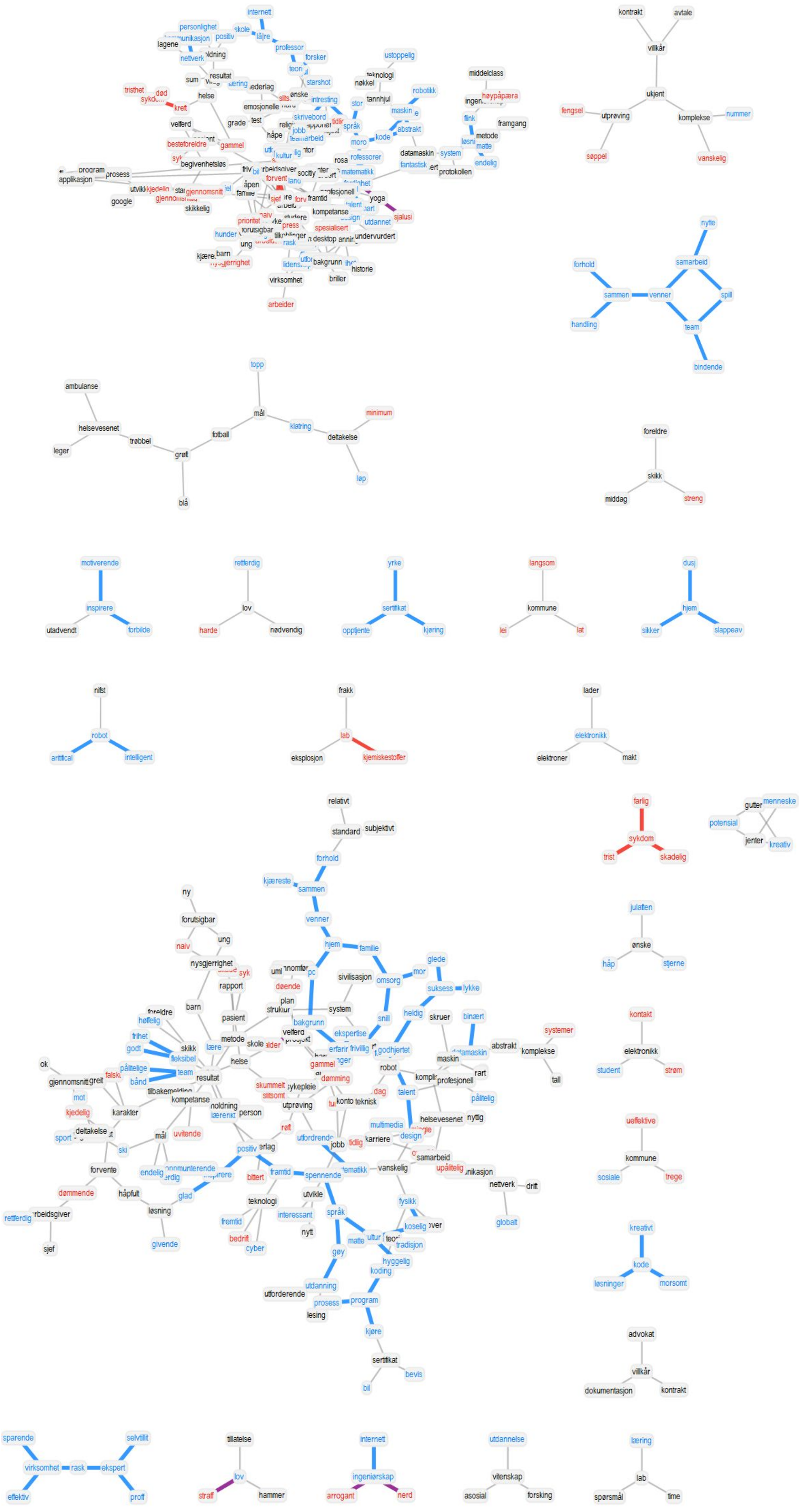

kontrakt
avtale
villkår
ukjent
fengsel
utprøving
søppel
komplekse
nummer
vanskelig
nytte
forhold
samarbeid
sammen
venner
spill
handling
team
bindende
topp
ambulanse
mål
minimum
helsevesenet
klatring
fotball
deltakelse
leger
trøbbel
grøt
løp
blå
foreldre
skikk
middag
streng
motiverende
inspirere
utadvendt
forbilde
rettferdig
lov
harde
nødvendig
yrke
sertifikat
opptjente
kjøring
langsom
kommune
lei
lat
dusj
hjem
sikker
slappeav
nifst
robot
artifical
intelligent
frakk
lab
eksplosjon
kjemiskestoffer
lader
elektronikk
elektroner
makt
farlig
sykdom
trist
skadelig
gutter
menneske
potensial
jenter
kreativ
julaften
ønske
håp
stjerne
kontakt
elektronikk
student
strøm
uaffektive
kommune
sosiale
trege
kreativ
kode
løsninger
morsomt
advokat
villkår
dokumentasjon
kontrakt
sparende
selvtillit
virksomhet
rask
ekspert
effektiv
proff
tillatelse
lov
straff
hammer
internett
ingeniørskap
arrogant
nerd
utdannelse
vitenskap
asosial
forsking
læring
lab
spørsmål
time

Figure S5

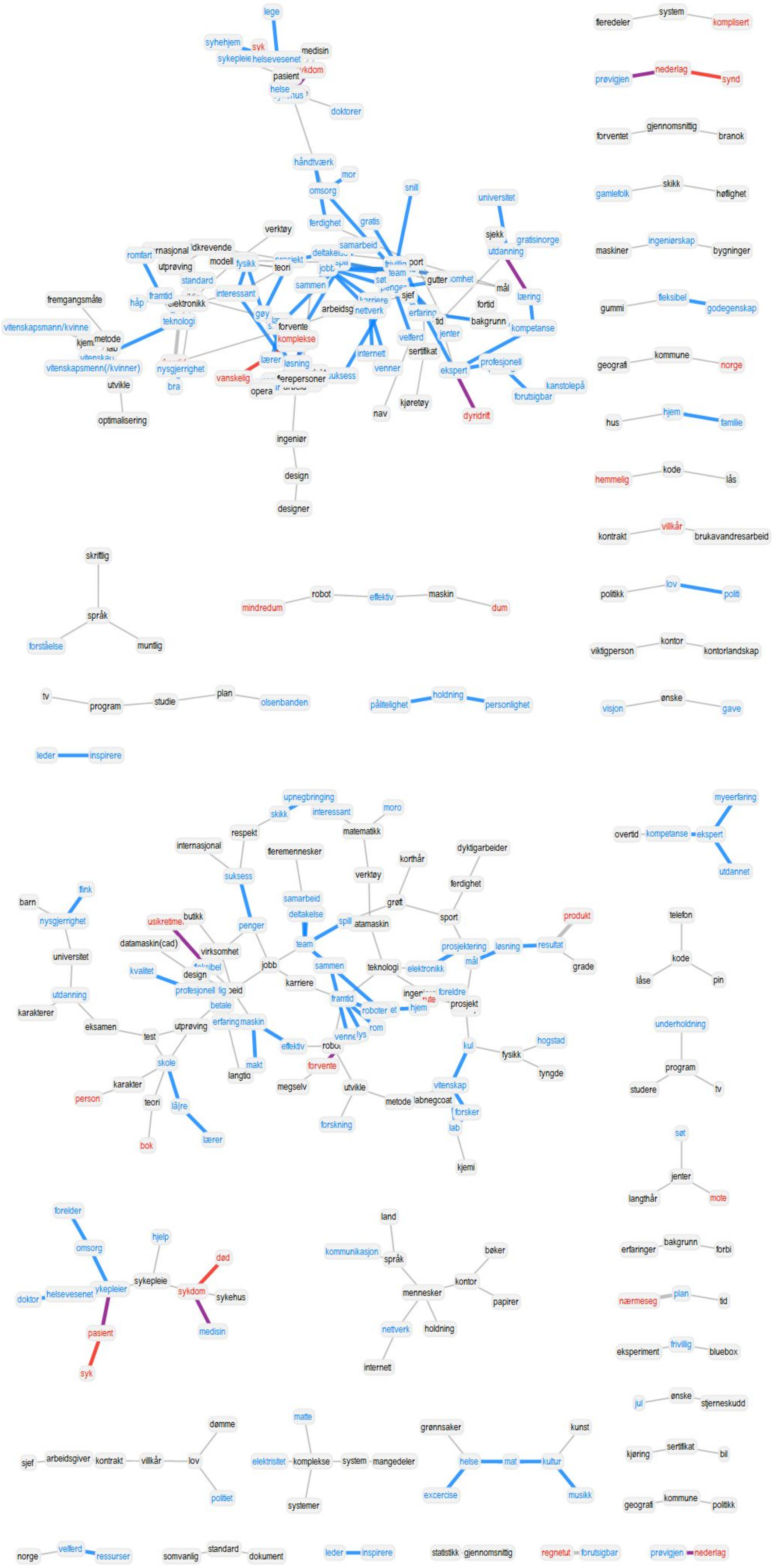

lege
syhehjem
syk
medisin
helsevesenet
pasient
helse
doktorer
håndtværk
mor
omsorg
snill
universitet
ferdighet
gratis
verktøy
samarbeid
sjekk
gratisnorge
utdanning
romfart
utprøving
modell
fysikk
teori
standard
gutter
mål
læring
fremgangsmåte
håp
framtid
interessant
sammen
arbeidsg
nettverk
fortid
bakgrunn
vitenskapsmann/kvinne
teknologi
gøy
forvente
erfaring
tid
kompetanse
metode
komplekse
velferd
jenter
vitenskapsmenn(/kvinner)
nysgjerrighet
lærer
løsning
internett
sertifikat
ekspert
profesjonell
bra
vanskelig
venner
kanstolepå
utvikle
forutsigbar
optimalisering
nav
kjøretøy
dyridrift
ingeniør
design
designer
fleredeler
system
komplisert
prøvigjen
nederlag
synd
forventet
gjennomsnittig
branok
gamlefolk
skikk
høflighet
maskiner
ingeniørskap
bygninger
gummi
fleksibel
godegenskap
geografi
kommune
norge
hus
hjem
familie
hemmelig
kode
lås
kontrakt
villkår
brukavandresarbeid
skriftlig
robot
effektiv
maskin
mindredum
dum
politikk
lov
politi
språk
forståelse
muntlig
viktigperson
kontor
kontorlandskap
tv
program
studie
plan
olsenbanden
pålitelighet
holdning
personlighet
visjon
ønske
gave
leder
inspirere
upnegbringing
skikk
interessant
moro
myeerfaring
respekt
matematikk
internasjonal
fleremennesker
dyktigarbeider
overtid
kompetanse
ekspert
utdannet
suksess
korthår
verktøy
ferdighet
flink
samarbeid
grøt
barn
deltakelse
sport
produkt
nysgjerrighet
usikretime
butikk
penger
spill
atamaskin
telefon
team
prosjektering
løsning
resultat
datamaskin(cad)
virksomhet
universitet
mål
sammen
teknologi
elektronikk
kode
kvalitet
design
jobb
grade
låse
pin
utdanning
profesjonell
karriere
foreldre
betale
framtid
prosjekt
roboter
hjem
karakterer
erfaring
maskin
eksamen
test
utprøving
underholdning
rom
effektiv
robot
hogstad
skole
kul
fysikk
program
makt
forvente
tyngde
langtid
karakter
megselv
utvikle
vitenskap
studere
person
teori
metode
labnegcoat
forsker
låre
forskning
lab
bok
lærer
søt
kjemi
jenter
langthår
mote
forelder
land
omsorg
hjelp
erfaringer
bakgrunn
forbi
kommunikasjon
bøker
død
språk
sykepleie
kontor
nærmeseg
plan
tid
doktor
helsevesenet
sykepleier
sykdom
sykehus
mennesker
papirer
pasient
medisin
nettverk
holdning
eksperiment
frivillig
bluebox
syk
internett
jul
ønske
stjerneskudd
dømme
matte
grønnsaker
kunst
sertifikat
kjøring
bil
sjef
arbeidsgiver
kontrakt
villkår
lov
elektrisitet
komplekse
system
mangedeler
helse
mat
kultur
politiet
systemer
excercise
musikk
geografi
kommune
politikk
standard
norge
velferd
ressurser
somvanlig
dokument
leder
inspirere
statistikk
gjennomsnittig
regnetut
forutsigbar
prøvigjen
nederlag

Figure S6

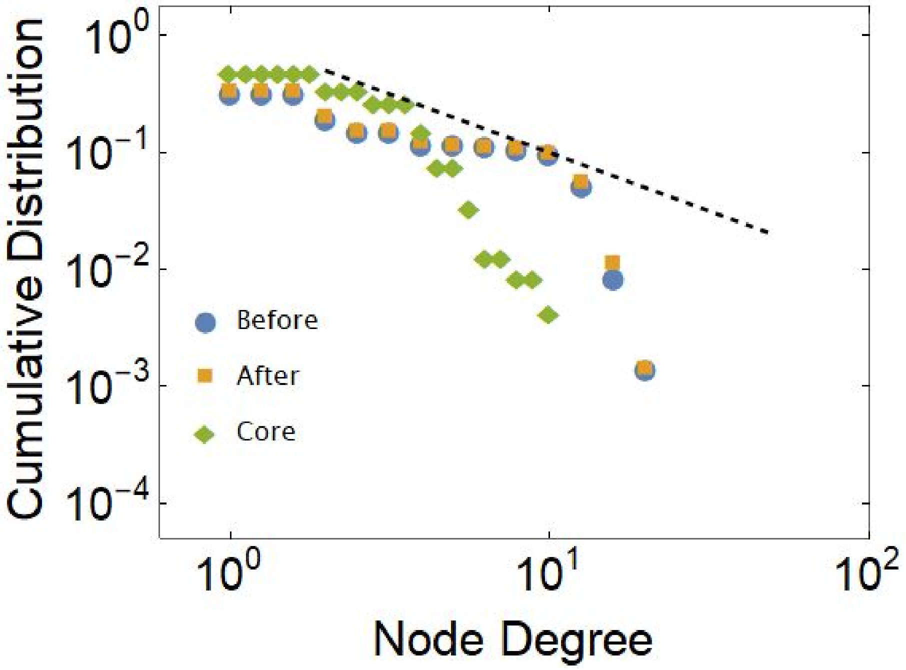

Figure S7

Supplementary Text S1 - Description of the Intervention Event

All the participants to the current study took part in a summer job experience taking place at i4Helse, University of Agder, Norway, from June to August 2019. i4Helse is an innovation hub aiming at facilitating and accelerating implementation of health and welfare solutions of tomorrow. One of its key missions is competence building in the upcoming generation of professionals in health and homecare, as well as in specialized healthcare provision, technological design and practical use of emerging technologies in the domain. Involvement of the University of Agder in the operation of i4Helse provides those synergies - of non-STEM and STEM skills students, whose forma mentis are explored, for the purposes of further application of the results for acceleration of synergic cooperation processes at i4Helse. The process of making the students to cooperate for building a “homecare assistant” robot began in spring 2019, in collaboration between i4Helse and a student-driven startup Young Industrial Innovators, was supported by the Mechatronic Innovation Lab, with tight involvement of municipal professionals, knowledgeable in so-called Early Identification of Life-threatening Conditions (Tidlig Identifisering av Livstruende Tilstander, or TILT, in Norwegian). The students came from the Faculty of Technology and Natural Sciences and Faculty of Health and Sports, including interns at Mechatronics Innovation Lab and the Center for Artificial Intelligence Research, University of Agder, Norway. The students completed a task relative to designing and assembling a mobile assisting TILT robot. Supervised by a municipal employee with practical knowledge in TILT and a couple of STEM professionals, the selected students in health and homecare and students in programming, engineering and robotics cooperated over the whole summer in order to fulfill this task. The students used equipment such as raspberry pi embedded data systems, with ports to connect with cameras, microphones for voice and language recognition, screens, sensors, bluetooth, mechanic sphygmomanometers, and robotic platforms for physical following human in movement. Research on changes in the forma mentis in students was conducted by Complex Science Consulting during short research stayings at the beginning and at the end of the summer job experience.

Supplementary Text S2 - Qualitative Feedback from Students

After the completion of the second cognitive experiment, at the end of their summer job experience, students were invited to provide feedback about their overall experience. Feedback sessions were held right after the completion of the cognitive experiment but without providing preliminary results to students. Feedback was provided through a guided dialogue, under the supervision of the first author. Students were asked to answer simple questions in the following order:

1. Do you think you achieved the main aims of the summer job experience?
2. Are you happy about the outcomes of the summer job?
3. What was your role in achieving these outcomes?
4. Which were the best and the worst elements of the summer job?
5. Which were the most useful skills that you acquired during this experience?

All the students considered the summer job a success in terms of achieving the aim of constructing an e-health robot. At the same time, all the students enjoyed the summer job event and provided positive feedback about it. Engineering students declared their involvement mostly in terms of practical tasks, related to the assembly of the robot and to its programming. Nursing students identified themselves as providing feedback to engineering students about the necessary features of a robotic assistant, based on their own personal nursing training. Nursing students did not actively code but followed the progress of engineering students, instead. Five out of six students indicated “communication” and “teamwork” as the key and most appreciated elements of the summer job experience. All students confirmed individually the roles of nursing and engineering students and the relevance of multidisciplinary interactions for progressing, especially in the last few weeks of work. Four students highlighted the last weeks as “hectic” and “very busy”. Despite the resulting stress, students expressed their satisfaction in completing the project on time. The most useful skill acquired during the summer job resulted being the capability of effectively discussing ideas across different backgrounds, enjoying personal interactions and collaborative working for professional growth. One engineering student and two nursing students also expressed their desire to improve their technical skills related to coding and programming after seeing the outcomes of the summer job.

Supplementary Table S1 - List of cue words

**arbeid**	**erfaring**	**ekspert**	**lov**
**utdanning**	**omsorg**	**datamaskin**	**ferdighet**
**ingeniørskap**	**fysikk**	**sykepleie**	**suksess**
**matematikk**	**teknologi**	**helse**	**vitenskap**
**nederlag**	**system**	**inspirere**	**utvikle**
**pasient**	**prosjekt**	**plan**	**resultat**
**forutsigbar**	**karriere**	**sykdom**	**gjennomsnittig**
**karakter**	**helsevesenet**	**nysgjerrighet**	**program**
**skikk**	**frivillig**	**ønske**	**utprøving**
**fleksibel**	**elektronikk**	**lære**	**sertifikat**
**kommune**	**hjem**	**jobb**	**arbeidsgiver**
**profesjonell**	**kode**	**villkår**	**standard**
**språk**	**kontor**	**holdning**	**maskin**
**robot**	**lab**	**løsning**	**test**
**nettverk**	**velferd**	**deltakelse**	**team**
**samarbeid**	**sammen**	**bakgrunn**	**teori**
**design**	**forvente**	**framtid**	**metode**
**virksomhet**	**kultur**	**komplekse**	**kompetanse**
**mål**	**gutter**	**jenter**	
